# Diffusion Acceleration with Gaussian process Estimated Reconstruction (DAGER)

**DOI:** 10.1002/mrm.27699

**Published:** 2019-03-01

**Authors:** Wenchuan Wu, Peter J. Koopmans, Jesper L.R. Andersson, Karla L. Miller

**Affiliations:** ^1^ Wellcome Centre for Integrative Neuroimaging, FMRIB, Nuffield Department of Clinical Neurosciences University of Oxford Oxford United Kingdom; ^2^ Erwin L. Hahn Institute for Magnetic Resonance Imaging University of Duisburg‐Essen Essen Germany; ^3^ High Field and Hybrid MR Imaging University Hospital Essen, University of Duisburg‐Essen Essen Germany

**Keywords:** diffusion MRI, Gaussian processes, image acceleration, k‐q reconstruction, SMS

## Abstract

**Purpose:**

Image acceleration provides multiple benefits to diffusion MRI, with in‐plane acceleration reducing distortion and slice‐wise acceleration increasing the number of directions that can be acquired in a given scan time. However, as acceleration factors increase, the reconstruction problem becomes ill‐conditioned, particularly when using both in‐plane acceleration and simultaneous multislice imaging. In this work, we develop a novel reconstruction method for in vivo MRI acquisition that provides acceleration beyond what conventional techniques can achieve.

**Theory and Methods:**

We propose to constrain the reconstruction in the spatial (k) domain by incorporating information from the angular (q) domain. This approach exploits smoothness of the signal in q‐space using Gaussian processes, as has previously been exploited in post‐reconstruction analysis. We demonstrate in‐plane undersampling exceeding the theoretical parallel imaging limits, and simultaneous multislice combined with in‐plane undersampling at a total factor of 12. This reconstruction is cast within a Bayesian framework that incorporates estimation of smoothness hyper‐parameters, with no need for manual tuning.

**Results:**

Simulations and in vivo results demonstrate superior performance of the proposed method compared with conventional parallel imaging methods. These improvements are achieved without loss of spatial or angular resolution and require only a minor modification to standard pulse sequences.

**Conclusion:**

The proposed method provides improvements over existing methods for diffusion acceleration, particularly for high simultaneous multislice acceleration with in‐plane undersampling.

## 1 INTRODUCTION

As neuroscience studies aim for higher spatial and angular resolution, image acceleration is an increasingly important part of the diffusion MRI (dMRI) toolkit. The acquisition time of a dMRI sequence is determined by the number of diffusion volumes (q‐space samples) and the scan time for each volume (in k space). The number of diffusion directions is critical for dMRI methods that benefit from high angular resolution (e.g., tractography). dMRI data are typically acquired using 2D echo planar imaging (EPI). While EPI is quite time efficient, dMRI sequences are overall very inefficient because ≥50% of the sequence time is typically dedicated to diffusion preparation. This inefficiency is compounded by the sequential slice acquisition, such that the scan time per volume is proportional to the number of slices. For fixed coverage, higher spatial resolution requires more slices and, therefore, increased scan time. When scan time is limited, there is a trade‐off between spatial coverage, spatial resolution, and angular resolution (density of q‐space sampling).

Recently, acquisition and reconstruction methods have been developed to mitigate this trade‐off. One major improvement is simultaneous multislice (SMS) imaging,^1^, ^2^, ^3^ which can reduce the volume acquisition time, enabling more diffusion directions within the same scan time. SMS techniques have particularly benefitted from improved k‐space encoding schemes like blipped‐CAIPI.^3^ However, due to the intrinsically low signal‐to‐noise ratio (SNR) of dMRI data and the ill‐conditioning at higher multiband (MB) factor, slice acceleration at 4× or above is still difficult to achieve. Moreover, SMS accelerations rely on the same coil hardware used to achieve in‐plane acceleration,^4^, ^5^ which is crucial at high spatial resolution to reduce EPI distortion and blurring. The combination of in‐plane and SMS accelerations is thus highly desirable, but currently ill‐conditioned. Regularized parallel imaging methods could potentially alleviate this problem by enforcing some prior information in the spatial domain.^6^, ^7^, ^8^, ^9^, ^10^ Although undersampling factors above 10 have been reported in structural or functional MRI, high acceleration is considerably more challenging in dMRI. For example, the Human Connectome Project (HCP) exhaustively evaluated SMS accelerations for both functional MRI and dMRI. The optimized HCP 3T protocol used MB = 3 (without in‐plane undersampling) for dMRI, compared with MB = 8 for functional MRI.^11^

An alternate approach is to accelerate in q‐space. Compressed sensing has been used to improve the estimation of diffusion profiles using a small number of q‐space samples and estimating the diffusion signal at unseen locations in q‐space.^12^, ^13^, ^14^, ^15^, ^16^, ^17^ These approaches exploit the information redundancy in q‐space and impose a sparsity constraint under a data transform (e.g., wavelet, total variation) or in a simple model space (e.g., summation of tensors).

Finally, a series of methods have proposed joint k‐q acceleration, with data undersampling in both k space and q space.^18^, ^19^, ^20^, ^21^ These methods all cast joint k‐q acceleration as a compressed sensing problem and differ primarily on the specific sparsifying transform or model. Most of these methods focus on in‐plane undersampling to enable comparable scan time to single‐shot acquisitions but at much lower distortion for a given resolution.

In this work, we propose a new method to accelerate dMRI acquisition by incorporating sharable information between q‐space samples. The smoothness of signal in q‐space has been exploited for acceleration.^20^ Gaussian processes (GPs)^22^ offer a general framework for smoothing and interpolating signals in a model‐independent manner. In diffusion imaging, this has been used to correct for eddy‐current induced distortions and subject movement^23^, ^24^ and replace outliers.^25^

Building on these approaches, we have integrated GPs into joint k‐q reconstruction to achieve high acceleration in the context of both in‐plane and slice‐wise acceleration. We call this approach Diffusion Acceleration with Gaussian process Estimated Reconstruction (DAGER). In this work, we focus on k‐q reconstruction of single‐shell dMRI data and more generalized q‐space sampling (i.e., multiple shells) is not covered. We use a Bayesian framework that learns the smoothness hyper‐parameters from the data, with no need for manually tuning reconstruction parameters.^22^ We demonstrate this approach using numerical simulations and in vivo acquisitions. Two acceleration schemes are evaluated: a very high in‐plane undersampling factor (exceeding the theoretical limit of conventional parallel imaging) and using a high SMS acceleration factor with in‐plane undersampling (the primary intended application). Both simulations and in vivo results demonstrate superior performance of DAGER compared with conventional parallel imaging methods. These improvements are achieved without loss of spatial or angular resolution and require only a minor modification to standard pulse sequences.

## 2 THEORY

### 2.1 GP modeling of dMRI signal

A GP is a statistical model defined as a probability distribution over functions where function values evaluated at any arbitrary set of input points have a joint Gaussian distribution.^22^ Consider dMRI as the output of a real‐valued function $S(q_{\text{i}}).$ The input $q_{\text{i}}$ is specified by a diffusion encoding direction and a b value. With GP modeling, any finite collection of dMRI signals u=S(q) at an input set $q=\left[q_{1},q_{2},\cdots ,q_{{\text{N}}_{\text{d}}}\right]$ follows a multivariate Gaussian distribution:

(1) u∼N(μ,Σ)

which is fully specified by a mean vector μ (N_d_×1) and a covariance matrix Σ (N_d_×N_d_). **u** (N_d_×1) contains dMRI signal from a single voxel for all diffusion volumes. N_d_ is the number of diffusion volumes. The spatial distribution of signals (i.e., covariance between voxels) is temporarily ignored for simplicity of notation, but will be covered in the “dMRI reconstruction with a GP prior constraint” section.

The GP covariance is modeled by a covariance function *C*, which is typically characterized by a small number of hyper‐parameters Θ. The dMRI signal is usually acquired on 1 or several q‐space shells, each corresponding to 1 b‐value. The spherical covariance function^26^ has been shown to have good agreement with the covariance observed from real dMRI data.^24^ For single‐shell acquisition, the corresponding spherical covariance function is given by:

(2) $$\begin{array}{c} C_{\theta }\left(\theta_{i,j};a\right)=\left\{\begin{array}{c} \begin{array}{c} \begin{array}{cc} 1-\frac{3\theta_{i,j}}{2a}+\frac{\theta_{i,j}^{3}}{2a^{3}} & \text{if}\;\theta \leq a \end{array}\\ \; \end{array}\\ \begin{array}{cc} \;0 & \;\text{if} \end{array}\;\theta >a \end{array}\right. \end{array}$$

Here, $\theta_{i,j}$ denotes the angle between the 2 vectors, $g_{i}$ and $g_{j}$, pointing from the q‐space origin to the q‐space points $q_{i}$ and $q_{j}$, defined as $\theta_{i,j}=\cos^{-1}\left\langle g_{i},g_{j}\right\rangle$. A smaller θ is associated with a larger covariance between the 2 q‐space points. The smoothness hyper‐parameter a governs the decay rate of the covariance with respect to θ and serves as an angular threshold.

For real dMRI data, 2 other hyper‐parameters, λ and σ, are also included in the covariance function: $C\left(q_{i},q_{j}\right)=\lambda C_{\theta }\left(\theta_{i,j};a\right)+\sigma^{2}\delta_{i,j}$, where λ captures the signal variability and $\sigma^{2}$ represents the noise variance that is uncorrelated in q‐space. $\delta_{i,j}$ is the Kronecker delta function. The covariance matrix can be derived accordingly

(3) $$\Sigma_{y}=\Sigma +\sigma^{2}I$$

with Σ defined as $\Sigma_{i,j}=\lambda C_{\theta }\left(\theta_{i,j};a\right)$, capturing the q‐space smoothness. **I** is an identity matrix.

If prior information about the mean signal for each diffusion direction is available, the mean function can also be modelled explicitly.^22^ Otherwise, the mean of a GP model is usually set to zero. Note a zero‐mean prior is not a strict limitation, as the posterior mean is not constrained to zero.

### 2.2 GP prediction of dMRI signal

GP methods allow us to place a prior distribution directly on the functions, which is transformed to a posterior distribution after having been fit to data. This posterior can be used to predict unseen dMRI signal, which has been used in data analysis for removing distortion and discarding outliers.^23^, ^25^ In the context of image reconstruction, it can be used to fill in missing data to accelerate acquisition.

Assume we have a set of dMRI images $y={\left[y\left(q_{1}\right),y\left(q_{2}\right),\cdots ,y\left(q_{{\text{N}}_{\text{d}}}\right)\right]}^{\text{T}}$, which contain additive noise:

(4) $$\begin{array}{c} y\left(q_{i}\right)=u\left(q_{i}\right)+n_{i} \end{array}$$

The noise $n_{i}$ is assumed to follow an independent and identically distributed (i.i.d.) Gaussian with zero mean and variance $\sigma_{\text{im}}^{2}$. The likelihood is:

(5) $$\begin{array}{c} p\left(y|u,q\right)=\prod_{i=1}^{{\text{N}}_{\text{d}}}\frac{1}{\sqrt{2\pi \sigma_{\text{im}}^{2}}}\exp\left(-\frac{{\left(y\left(q_{i}\right)-u\left(q_{i}\right)\right)}^{2}}{\sigma_{\text{im}}^{2}}\right) \end{array}$$

If we cast our estimation of u in terms of Bayes’ theorem with a GP prior (Equation 1), the maximum a posteriori (MAP) estimation provides a point estimate^27^:

(6) $$\overset{¯}{u}=\underset{u}{\text{arg}\;\text{min}}\left(\frac{1}{2\sigma_{\text{im}}^{2}}||u-{y||}_{2}^{2}+\frac{1}{2}{\left(u-\mu \right)}^{\text{H}}\Sigma^{-1}\left(u-\mu \right)\right)$$

whose analytical solution is given by $\overset{¯}{u}={\left(\sigma_{\text{im}}^{-2}I+\Sigma^{-1}\right)}^{-1}\left(\sigma_{\text{im}}^{-2}y+\Sigma^{-1}\mu \right)$, which is a linear combination of prior and data with weights determined by the prior covariance and noise variance.

### 2.3 dMRI reconstruction with a GP prior constraint

The previous sections have described GPs in terms of a single voxel. In image reconstruction, we acquire data in k space and estimate the unknown signal in image space. The dMRI measurement can be described as a linear model:

(7) d=Au+n

where **u** (N_v_N_d_×1) is extended to contain the signals for all diffusion volumes from all voxels. N_v_ is the number of voxels. **d** (N_v_N_d_N_c_×1) denotes the acquired k‐space data for all N_d_ diffusion volumes. Note the vector **y** in the previous section is the observation in image space. N_c_ is the number of coils. **A** (N_v_N_d_N_c_× N_v_N_d_) is the system matrix combining sensitivity encoding, Fourier transform and the k‐space sampling operation. **n** (N_v_N_d_N_c_×1) is the measurement noise, which is assumed to follow an i.i.d. Gaussian distribution with zero mean and variance $\sigma_{\text{k}}^{2}$.

We now extend the GP model (Equation 1) to include the spatial distribution of signals

(8) $$\begin{array}{c} u∼N\left(\mu ,\Sigma ⊗I_{{\text{N}}_{\text{v}}}\right) \end{array}$$

where μ and Σ have the size of N_v_N_d_×1 and N_d_×N_d_, respectively. $I_{{\text{N}}_{\text{v}}}$ is a N_v_×N_v_ identity matrix. ⊗ is the Kronecker product. The full covariance matrix (N_v_N_d_×N_v_N_d_) is:

(9) $$\begin{array}{c} \Sigma ⊗I_{{\text{N}}_{\text{v}}}=\left[\begin{array}{cc} \begin{array}{cc} \Sigma_{1,1}I_{{\text{N}}_{\text{v}}} & \Sigma_{1,2}I_{{\text{N}}_{\text{v}}}\\ \Sigma_{2,1}I_{{\text{N}}_{\text{v}}} & \Sigma_{2,2}I_{{\text{N}}_{\text{v}}} \end{array} & \begin{array}{cc} \ldots & \Sigma_{1,{\text{N}}_{\text{d}}}I_{{\text{N}}_{\text{v}}}\\ \ldots & \Sigma_{2,{\text{N}}_{\text{d}}}I_{{\text{N}}_{\text{v}}} \end{array}\\ \begin{array}{cc} \vdots & \vdots \\ \Sigma_{{\text{N}}_{\text{d}},1}I_{{\text{N}}_{\text{v}}} & \Sigma_{{\text{N}}_{\text{d}},2}I_{{\text{N}}_{\text{v}}} \end{array} & \begin{array}{cc} \ldots & \vdots \\ \ldots & \Sigma_{{\text{N}}_{\text{d}},{\text{N}}_{\text{d}}}I_{{\text{N}}_{\text{v}}} \end{array} \end{array}\right] \end{array}$$

The covariance values are purely a function of angular distance between directions (i.e., Equation 2). Although each pair of directions may have a unique covariance value $\Sigma_{\text{i},\text{j}}$, there is only 1 set of hyper‐parameters to estimate.

Conventional parallel imaging aims to find the maximum likelihood estimate (i.e., the point estimate that does not include any prior constraints)^28^:

(10) $$\begin{array}{c} u=\underset{u}{\text{arg min}}||\mathrm{Au}-{d||}_{2}^{2} \end{array}$$

By incorporating a GP prior on the signal, the MAP estimate can be obtained as^27^:

(11) $$\begin{array}{c} u=\underset{u}{\text{arg min}}\left(\frac{1}{2\sigma_{\text{k}}^{2}}||\mathrm{Au}-{d||}_{2}^{2}+\frac{1}{2}{\left(P^{\text{H}}u-\mu \right)}^{\text{H}}{\left(\Sigma ⊗I_{{\text{N}}_{\text{v}}}\right)}^{-1}\left(P^{\text{H}}u-\mu \right)\right) \end{array}$$

Disregarding the P matrix (discussed below), this is a straightforward extension of the MAP estimation given in Equation 6. This MAP formulation resembles standard linear image reconstructions with a regularization between 2 cost functions. The unusual feature here is that the regularization parameter ($\sigma_{\text{k}}^{2}$ and Σ) is clearly defined based on the Bayesian likelihood, as discussed in the next section.

P denotes the motion‐induced phase errors and H is the conjugate transpose operator. The phase errors are primarily driven by bulk and cardiac motion during the diffusion preparation and have no relation to the q‐space locations. The acquired complex signal thus violates the GP assumption that information is correlated in local q‐space. $P^{\text{H}}$ is included in the reconstruction to realign the image phase across diffusion volumes. The phase errors can be measured from a low‐resolution navigator^29^, ^30^ or estimated from the imaging data.^31^

### 2.4 GP hyper‐parameter estimation

Solving Equation 11 requires knowledge of the hyper‐parameters $\Theta =\left[\lambda ,\text{a},\sigma \right]$ encapsulated in the covariance matrix Σ. Using Bayes theorem, the optimal hyper‐parameters can be estimated from image data by maximizing the marginal likelihood p(y|q,Θ). In practice, one typically minimizes the negation of the log of the marginal likelihood:

(12) $$\begin{array}{c} \Theta =\underset{\Theta }{\;\arg\;\min}\;\left(\frac{1}{2}y^{\text{T}}\Sigma_{y}^{-1}y+\frac{1}{2}\text{log}\left|\Sigma_{y}\right|+\frac{{\text{N}}_{\text{d}}}{2}\log2\pi \right) \end{array}$$

where $\left|\Sigma_{y}\right|$ is the determinant of $\Sigma_{y}.$

## 3 METHODS

### 3.1 Image reconstruction and hyper‐parameter estimation

An iterative scheme is used to jointly estimate the optimal hyper‐parameters and the underlying images: in each iteration, a set of hyper‐parameters is calculated (Equation 12) using the images reconstructed from the previous iteration; then a new image reconstruction is conducted with the updated hyper‐parameters (Equation 11). As the iteration goes on, it is expected both the hyper‐parameter estimation and the image reconstruction can be improved.

The noise variance estimated from the reconstructed images ($\sigma_{\text{im}}^{2}$) is different from the noise variance of the k‐space data ($\sigma_{\text{k}}^{2}$), thus one cannot directly use $\sigma_{\text{im}}^{2}$ in the MAP reconstruction (Equation 11). Nevertheless, the propagation of noise in parallel imaging can be quantitatively measured,^5^ which provides a way to derive $\sigma_{\text{k}}$ from $\sigma_{\text{im}}$.

The pipeline of the proposed method is summarized below:

**Initial**: Conventional sensitivity encoding technique (SENSE) is used to reconstruct an image initialization $u^{0}$.

$\sigma_{\text{k}}$ is estimated from $\sigma_{\text{im}}^{0}$ in 1000 randomly chosen brain voxels as:

(13) $$\begin{array}{c} \sigma_{\text{k}}=\frac{1}{1000}\sum_{i=1}^{1000}\frac{\sigma_{\text{im}}^{0}\left(\rho_{i}\right)}{g^{*}\left(\rho_{i}\right)}=\frac{1}{1000}\sum_{i=1}^{1000}\frac{\sigma_{\text{im}}^{0}\left(\rho_{i}\right)}{\sqrt{\;{\left[{\left(S^{\text{H}}\Phi S\right)}^{-1}\right]}_{\rho_{i},\rho_{i}}}} \end{array}$$

where $g^{*}$ is the noise propagation function, derived from the coil sensitivities S and the coil noise covariance matrix Φ.^5^ While the hyper‐parameter $\sigma_{\text{im}}$ is updated each iteration, $\sigma_{\text{k}}$ is calculated in the first iteration and fixed thereafter.

**Iteration n**: The optimal hyper‐parameters ($a^{n-1}$, ${\;\lambda }^{n-1}$,$\sigma_{\text{im}}^{n-1}$) are estimated from the signal across all diffusion directions extracted for 1000 randomly chosen brain voxels in $u^{\text{n}-1}$. The final estimates are an average of the estimates from all voxels. A covariance matrix $\Sigma^{n-1}$ is then constructed and the posterior mean ${\overset{¯}{u}}^{\text{n}-1}$ is calculated according to Equation 6.

Finally, we reconstruct all diffusion volumes using ${\overset{¯}{u}}^{\text{n}-1}$ and $\Sigma^{\text{n}-1}\;$as prior constraints:

(14) $$\begin{array}{c} u^{\text{n}}=\underset{u}{\text{arg min}}\left(\frac{1}{\sigma_{\text{k}}^{2}}||\mathrm{Au}-{d||}_{2}^{2}+{\left(P^{\text{H}}u-{\overset{¯}{u}}^{\text{n}-1}\right)}^{\text{H}}{\left(\Sigma^{\text{n}-1}⊗I_{{\text{N}}_{\text{v}}}\right)}^{-1}\left(P^{\text{H}}u-{\overset{¯}{u}}^{\text{n}-1}\right)\right) \end{array}$$

**Output**: We stop iterating when a fixed number of iterations is reached or the normalized solution update $\left|\right|u^{\text{n}}-u^{\text{n}-1}\left|\right|/\left|\right|u^{\text{n}-1}\left|\right|$ is below a preset threshold. The last reconstruction (Equation 14) will be the result.

### 3.2 k‐q sampling

**Figure 1:**
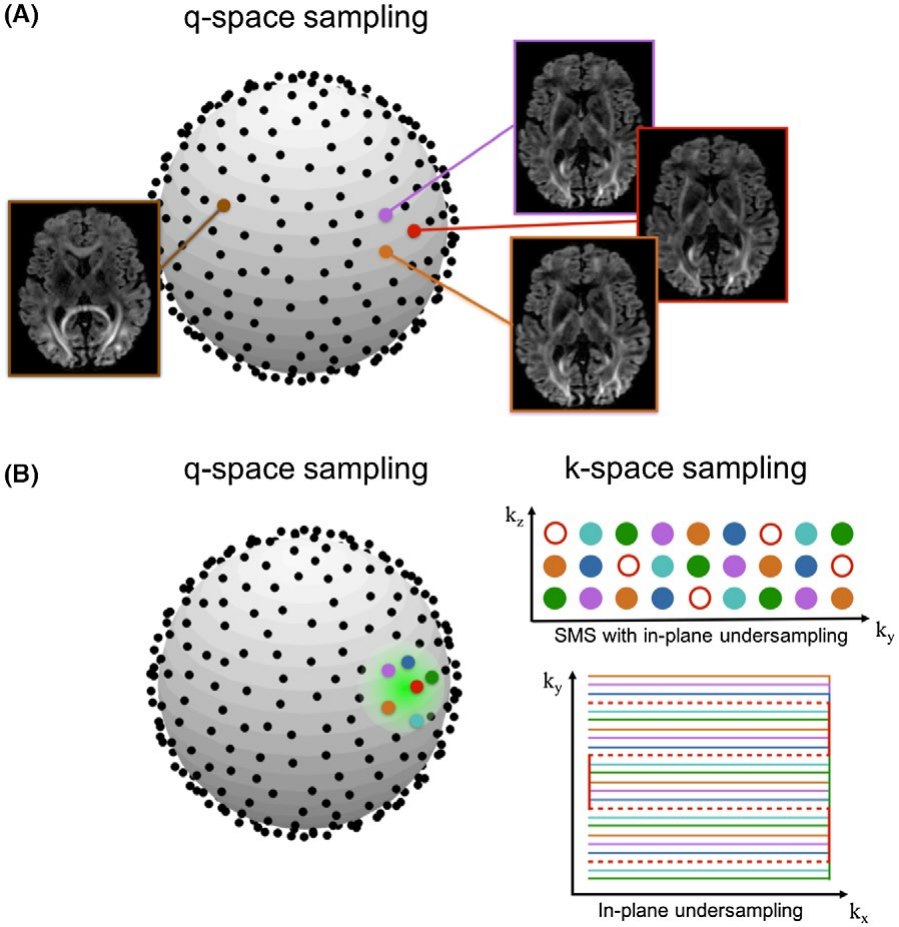
A, DAGER reconstruction takes advantage of local smoothness in q‐space: dMRI images that are near to each other in q‐space look very similar. B, The proposed k‐q sampling scheme: using different k‐space sampling patterns in the local q‐space neighborhood. Each color corresponds to a different sampling pattern in k‐space as shown on the right. Here, R = 6 is used for the in‐plane undersampling example (without k_z_ blips), and SMS = 3 with R = 2 is used for the SMS example. One set of k‐space sampling pattern is displayed as red circles (for SMS) and dashed lines (for in‐plane undersampling) for better visualization

Joint k‐q reconstruction could be improved by using different k‐space sampling patterns for different diffusion volumes.^19^, ^20^, ^21^ Smoothness in q‐space means nearby q‐space points share more common features (i.e., the images are more similar than distant points, Figure 1A). We explore the extent to which we can improve the DAGER reconstruction by using different k‐space undersampling patterns within a local q‐space neighborhood. Specifically, we use a graph model approach to design the proposed k‐q undersampling.

For EPI with an undersampling factor of R, there exist R unique k‐space sampling patterns that can be applied at different q‐space points, produced by shifting the undersampled trajectory along the phase‐encoding direction and slice direction (for SMS). For each q‐space location, a neighborhood is defined including itself and its R‐1 nearest neighbors. The task of assigning different k‐space sampling patterns for the q‐space points within a local neighborhood (Figure 1B) can be equivalently described as a graph‐coloring problem. In this formulation, each q‐space point corresponds to a vertex and an edge between 2 vertexes exists if the corresponding 2 q‐space points are within the same neighborhood. If the constructed graph can be colored using R different colors while obeying the constraint that no connected vertexes share the same color, the resultant coloring scheme provides the desired k‐q undersampling. Graph coloring is an “NP complete” problem, which means no efficient algorithms are available to provide the exact optimal solution. However, it also establishes an equivalence to all NP‐complete problems, for which several efficient algorithms exist. In this work, we applied a greedy algorithm to approximate the solution.^32^

### 3.3 Simulation

One common way to validate an acceleration method is to acquire fully sampled k‐space and retrospectively undersample it to generate simulation data sets. However, this approach is not very compatible with dMRI acquisition which suffers from shot‐to‐shot phase variation caused by subject motion. Instead, we derive simulation from a simplified signal model fitted voxel‐wise to high‐quality in vivo data. This is particularly helpful for evaluating the performance of DAGER with and without phase corruption. However, this simplified signal representation is by construction smooth in q‐space and is not perfectly representative of in vivo data. These simulations thus represent a well‐conditioned data environment, and ultimately it is crucial to perform in vivo evaluations.

Simulations were constructed based on dMRI data from the HCP, consisting of 3 shells (1000, 2000, and 3000 s/mm^2^) and 270 diffusion directions, which had been preprocessed to account for eddy currents and distortions.^33^ The data were fit with a ball‐and‐stick model,^34^, ^35^ which was used to generate simulated data based on the same model. We simulated in‐plane undersampling with a small number of channels, aimed to test the limits of the proposed method by using extreme acceleration along 1 dimension. We also conducted SMS simulations with in‐plane undersampling targeting the actual application we would like to achieve in practice. Therefore, the same number of channels were used for SMS simulation as for the in vivo data acquisition (the “In vivo experiments” section).

#### 3.3.1 In‐plane undersampling

Multichannel dMRI data were simulated with 128 directions, b = 1000 s/mm^2^ and an 8‐channel head coil (https://www.ismrm.org/mri_unbound/simulated.htm). Complex random noise was added to the data. Three in‐plane undersampling factors R = 4,6,10 were evaluated, with R = 10 exceeding the theoretical parallel imaging limits for the simulated 8‐channel coil. Simulations were generated with and without acquisition‐to‐acquisition phase errors. The phase errors consisted of a constant offset randomly selected between [‐π, π] and a linear phase term in image space, corresponding to a shift along k_y_ by a random distance between [0, 2Δk_y_]. Δk_y_ is the phase‐encoding step size. The effects of nonlinear phase errors are demonstrated in the in vivo experiments.

#### 3.3.2 SMS with in‐plane undersampling

An SMS data set with 4‐fold acceleration (MB = 4) was simulated with 192 directions, b = 1000 s/mm^2^ and 31.5‐mm slice gap, in‐plane undersampling factor R = 3, blipped‐CAIPI^3^, ^36^ with a field of view (FOV)/4 interslice image shift. The total undersampling factor was 12. Coil sensitivity maps were measured using a 32‐channel coil. Noise and phase errors were added to the data as described in the previous section.

### 3.4 In vivo experiments

Four subjects were scanned on a Siemens 7T scanner. Informed consent in accordance with local ethics was obtained before each scan. SMS data were acquired in all 4 subjects, while additional in‐plane undersampling scans were performed on 1 subject.

#### 3.4.1 Sequence implementation

A 2D spin‐echo dMRI sequence was modified to include SMS acquisition and the blipped‐CAIPI encoding scheme.^3^ After each imaging echo, a 2D navigator can optionally be acquired following a second refocusing pulse. Two k‐q sampling strategies were implemented: “fixed” sampling with the same k‐space lines acquired at each q‐space location, and “variable” sampling using the graph‐coloring algorithm described above to sample different k‐space locations in a local q‐space neighborhood. Coil sensitivities were measured using the FLEET‐ACS method.^37^, ^38^ For in‐plane undersampling acquisitions, a bipolar diffusion‐preparation^39^ scheme was used to reduce eddy current effects and a 64 × 64 navigator was acquired.

A major challenge for SMS dMRI at 7T is specific absorption rate. Three aspects of the sequence implementation (including radiofrequency pulses, diffusion preparation, and navigator acquisition) were optimized to reduce specific absorption rate for SMS acquisitions (see Supporting Information Methods S1, which is available online).

#### 3.4.2 In‐plane undersampling

The dMRI data were acquired from subject 1 using a 32‐channel coil. Two acceleration factors R = 3,6 were acquired with 1.5 × 1.5 × 1.5 mm^3^ resolution (matrix size 168 × 168, 40 slices, b = 1000 s/mm^2^ and 128 directions, repetition time (TR)/echo time = 6500 ms/82 ms, total scan time 13.8 min). The R = 3 data were acquired with partial Fourier 6/8, which were used as reference for comparison to R = 6 data.

Navigator and b = 0 data were reconstructed using conventional SENSE, which might incur significant artifact at high acceleration factors. Hence, for the R = 6 imaging data, the navigator was undersampled by R = 4 to reduce artifacts while producing similar distortions as the imaging data; the b = 0 data were acquired using multishot EPI (6 segments). We acquired 5 b = 0 volumes, 1 of which had reversed phase‐encoding direction to enable correction of susceptibility induced distortion.

#### 3.4.3 SMS with in‐plane undersampling

SMS data were acquired at MB factor of 4 using blipped‐CAIPI with a FOV/4 interslice image shift. Twenty‐one slice sets with 31.5‐mm slice gap were acquired, resulting in a total of 84 slices (126 mm) for full brain coverage. In‐plane undersampling R = 3 was also applied, for a total undersampling factor of 12. Other scan parameters were 1.5 mm isotropic resolution, matrix size 156 × 156, TR/echo time = 3500 ms/71 ms, b = 1000 s/mm^2^, 192 diffusion directions, total scan time 11 min. Four sets of b = 0 data were acquired using multishot EPI (12 segments), including 1 volume with reversed phase‐encoding direction for distortion correction. The multishot sequence is sensitive to subject motion, which could be mitigated by using lower undersampling factors (Supporting Information Discussion S1).

Single‐band (SB) data were acquired from all subjects for comparison to SMS data. For the subjects 1‐3, a set of SB data was acquired with the same FOV (i.e., 84 slices) as the SMS protocol, which was used as a full‐FOV reference. For these scans, a longer TR (12 s) and fewer diffusion directions (i.e., 50) were used to achieve similar acquisition time. For subject 4, 3 repetitions of SB data were acquired using the same sequence parameters as in the SMS protocol but containing only 21 slices to match the TR. The acquisition time was 33 min. These data were used to provide a high‐SNR reference to identify the degree of angular blurring incurred by the DAGER reconstruction.

### 3.5 Reconstruction implementation

Images were reconstructed using both DAGER and conventional parallel imaging methods, specifically SENSE for in‐plane undersampling and SMS‐SENSE for slice acceleration, which were implemented with custom code.

The SMS‐SENSE implementation followed a 3D k‐space formulation^40^, ^41^ where in‐plane undersampling and SMS acquisition are treated as undersampling along k_y_ and k_z_ dimension. A 2D‐SENSE^42^, ^43^ method was used to reconstruct the data. Due to the ill‐conditioning of the reconstruction, Tikhonov regularization was used in SENSE and SMS‐SENSE.^40^ We used the L‐curve approach^44^ to find the optimal regularization parameter (Supporting Information Figure S1).

#### 3.5.1 Phase error estimation

In simulation, the phase was estimated using the central k‐space (32 × 32) of the imaging data, which provides perfect knowledge of the simulated motion (zeroth and first‐order phase).

For the non‐SMS in vivo data, for which a navigator was acquired, conventional SENSE was applied to reconstruct the navigator image. This was then smoothed by a 2D hamming filter to suppress ringing artifacts and noise. The phase of the image was used as an estimate of the phase errors. We refer to this approach as “navigator based.”

For SMS in vivo data, which we did not acquire a navigator, the phase errors were estimated from the imaging data.^31^ Images were first reconstructed using SMS‐SENSE, followed by a total‐variation de‐noising.^45^ In this work, we found the reconstruction with the optimal regularization parameter still contained some residual aliasing artifacts. As minimal aliasing is crucial for DAGER to achieve accurate phase estimate, a lower regularization parameter was used in this reconstruction (Supporting Information Figure S1). The reconstructed image was used as an initialization for DAGER reconstruction. The phase of the de‐noised image was used as the phase‐error estimate. We refer to this approach as “navigator free.”

#### 3.5.2 DAGER reconstruction

The symmetric property of the diffusion signal was considered in simulation, where the covariance function was calculated with θ defined as $\theta_{i,j}=\cos^{-1}\left|〈g_{i},g_{j}〉\right|$.^24^ For in vivo data, the signal symmetry was not incorporated to reduce the effects of distortion induced by eddy currents and/or Maxwell terms on DAGER reconstruction. This led to a definition of $\theta_{i,j}=\cos^{-1}〈g_{i},g_{j}〉$.

Hyper‐parameters were calculated (Equation 12) using the conjugate gradient method. To avoid negative values for signal variance and noise variance, the model was modified to estimate $e^{\lambda }$ and $e^{\sigma }$ rather than λ and σ : $C\left(q_{i},q_{j}\right)=e^{\lambda }C_{\theta }\left(\theta_{i,j};a\right)+e^{2\sigma }\delta_{i,j}$.

Iteration stopped when either the iteration number reached 20 or the normalized solution update ($\left|\right|u^{\text{n}}-u^{\text{n}-1}\left|\right|/\left|\right|u^{\text{n}-1}\left|\right|$) was below 0.002.

### 3.6 Analysis

#### 3.6.1 Simulation data

Normalized root‐mean‐squared‐error (NRMSE) values were calculated between the reconstructed images and the noise‐free ground‐truth with a brain mask to emphasize the reconstruction errors within the brain region.

#### 3.6.2 Tensor and tractography modeling

The in vivo data were first processed using Topup and Eddy^23^ to correct image distortions, and then fitted with a tensor model using DTIFIT^46^ and, separately, a Bayesian ball‐and‐stick model with 2 fiber populations within a single voxel (BEDPOSTX).^34^, ^35^ Probabilistic tractography of major white matter tracts was conducted using the AutoPtx tool in FSL (https://fsl.fmrib.ox.ac.uk/fsl/fslwiki/AutoPtx).^47^ AutoPtx defines 14 major white matter pathways in a predefined atlas that avoids any operator bias. Tractography was not applied to the SB data from subject 4 due to the limited FOV.

#### 3.6.3 Investigation of induced angular blurring

Because DAGER uses a GP‐based smoothness prior on q‐space, it can potentially induce angular blurring if it overestimates the smoothness. To evaluate the accuracy of fiber orientation, we compared the fiber orientations between the SMS images reconstructed using DAGER and SENSE and a high‐SNR, non‐SMS reference in subject 4. The reference data were constructed by averaging 2 repetitions SB data (SB‐2ave). The mean of the posterior distribution of the fiber orientations estimated was used for comparison. A mask was used to exclude fibers having a wide orientation distribution around the principle diffusion direction, which was generated from the “dispersion” map (BEDPOSTX output) using a threshold of 0.03. The angular differences for the 2 fiber populations were calculated between the SMS images and SB‐2ave, as well as between an independent set of SB data (SB‐1ave) and SB‐2ave.

#### 3.6.4 Investigation of number of directions

To use q‐space smoothness, there must be nearby q‐space samples that lie within the extent of this smoothness. We investigated the effect of the number of diffusion directions on the different reconstructions. The 192‐direction data set was sub‐sampled to 160, 128, and 64 directions. Finally, we investigated the effect of error in the smoothness hyper‐parameter estimation using the set of 64‐directions. Two additional SMS‐DAGER reconstructions were applied with the hyper‐parameter *a* fixed to 1 (to include ~12 points in each q‐space neighborhood) and π (to use all q samples) throughout the reconstruction, where the estimated value for *a* is 0.7 (having ~7 points in each q‐space neighborhood). NRMSE values for different reconstructions were calculated using SB‐2ave as a reference. Before calculation, images were registered to half‐way space using FMRIB’s Linear Image Registration Tool (FLIRT) with rigid body 6 DOF and correlation‐ratio cost function.^48^

Simulation and acquisition parameters, reconstruction algorithms and data analyses described above are summarized in Table 1.

**Table 1:** Summary of simulation and acquisition parameters, and the reconstruction algorithms and analysis methods used in data processing

	Acquisition										Reconstruction			Analysis		
	Subj. ID	Diff. dir.	b value (s/mm^2^)	MB/R	TR/TE (s/ms)	TA (min)	Nav.	No. of slices	Ave.	No. of coils	SENSE	SMS‐SENSE	DAGER	DTIFIT	BEDPOSTX	AutoPtx
*In vivo protocols*																
SMS with in‐plane undersampling	Subj. 1 2 3 4	192	1000	4/3	3.5/71	11	‐	21 slice group	1	32	‐	Y	Y	Y	Y	Y
Single‐band reference	Subj. 1 2 3	50	1000	‐/3	12/71	11	‐	84	1	32	Y	‐	‐	Y	Y	Y
	Subj. 4	192	1000	‐/3	3.5/71	33	‐	21	3	32	Y	‐	‐	Y	Y	‐
In‐plane undersampling	Subj. 1	128	1000	‐/3,6	6.5/82	13.8	Y	40	1	32	Y	‐	Y	Y	Y	‐
*Simulation protocols*																
SMS with in‐plane undersampling	‐	192	1000	4/3	5.5/89	‐	Y	1 slice group	1	32	‐	Y	Y	‐	‐	‐
In‐plane undersampling	‐	128	1000	‐/ 4, 6, 10	5.5/89	‐	Y	1	1	8	Y	‐	Y	‐	‐	‐

## 4 RESULTS

### 4.1 Simulations of in‐plane acceleration

**Figure 2:**
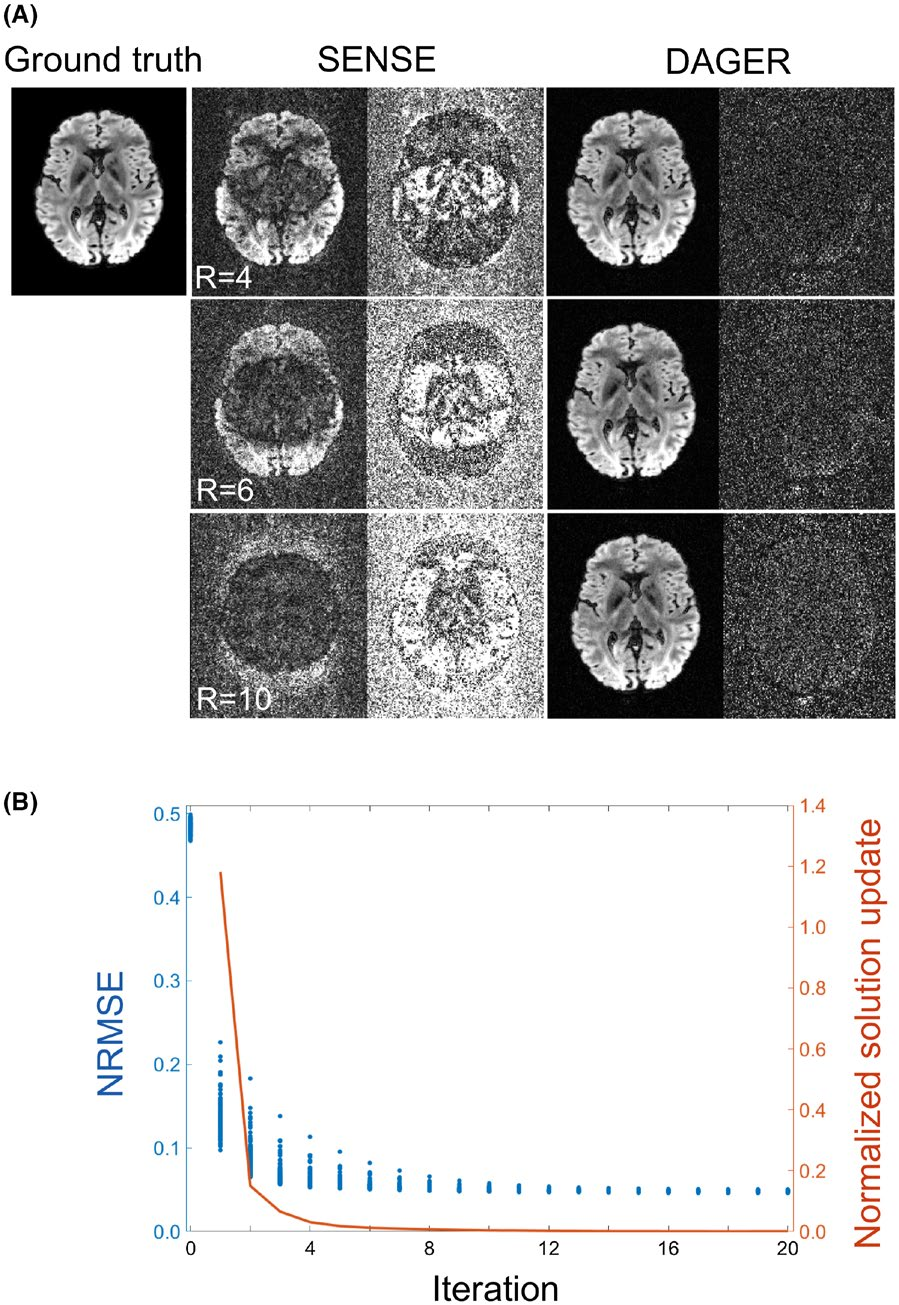
Simulation of in‐plane undersampling. A, Three undersampling factors R = 4, 6, and 10 are evaluated. Reconstructed images are shown on the left for each method. Difference from ground truth is shown on the right for each method, scaled by a factor of 3 for SENSE and 10 for DAGER. NRMSE values for SENSE are 0.36, 0.48, and 0.63 and for DAGER are 0.04, 0.05, and 0.06 (for R = 4, 6, 10, respectively). Note the simulations with R = 10 exceed the theoretical limit of conventional parallel imaging, but that nevertheless DAGER reconstruction manages to recover the underlying image with good fidelity. B, Convergence of the DAGER reconstruction for R = 6. Blue: NRMSE for all diffusion directions over 20 iterations (each point represents 1 diffusion direction). Red: Normalized solution update $\left|\right|u^{\text{n}}-u^{\text{n}-1}\left|\right|/\left|\right|u^{\text{n}-1}\left|\right|$ in 20 iterations. Note the iteration‐0 corresponds to the initial SENSE reconstruction

Figure 2A shows the reconstruction with R = 4, 6, and 10 using the variable k‐q sampling. With only 8 coil elements, conventional SENSE cannot reconstruct the images correctly for any undersampling factor. The reconstruction is substantially improved using DAGER, which reduces the NRMSE by a factor of ∼9 for the R = 10 acceleration compared with SENSE, with residuals appearing noise‐like rather than structured. Both the variable k‐q sampling scheme and phase‐error correction are important in the reconstruction (see Supporting Information Figure S2).

Figure 2B shows the convergence of the DAGER reconstruction with R = 6 data. The NRMSEs for all diffusion volumes decrease monotonically with iteration and reach a minimum after approximately 13 iterations. Although NRMSE is a useful metric to evaluate stopping criteria, ground truth is not available in practice. The normalized solution update has similar decay behavior to NRMSE and can be used to monitor the reconstruction progress. The reconstruction is stopped after 15 iterations.

DAGER reconstruction at different noise levels demonstrates image fidelity (as reflected in NRMSE) is not very sensitive to the estimated $\sigma_{\text{k}}$ for SNR ≥20 (Supporting Information Figure S3). Moreover, $\sigma_{\text{k}}$ is accurately estimated and reconstructed NRMSEs are close to optimal at SNR ≥10, which is crucial given that this parameter is not iteratively updated.

### 4.2 Simulations of SMS with in‐plane undersampling

**Figure 3:**
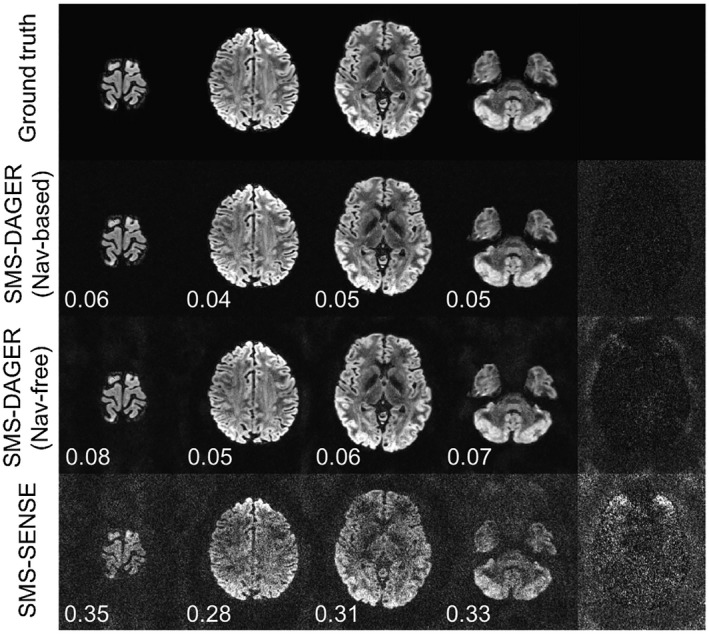
Simulation of SMS data with total acceleration of 12 (MB = 4 and in‐plane R = 3). Three reconstruction approaches are evaluated: SMS‐SENSE, SMS‐DAGER with navigator acquisition (Nav‐based), and SMS‐DAGER without navigator acquisition (Nav‐free). The 4 columns on the left are reconstructed images for the 4 simultaneously excited slices, with NRMSE values given on the bottom left (calculated for the brain region only). The rightmost column contains the difference images for the third slice, which are scaled by a factor of 5 for SMS‐SENSE and 10 for SMS‐DAGER

As shown in Figure 3, SMS‐DAGER achieves a considerably improved reconstruction compared with SMS‐SENSE (tailored SENSE reconstruction for SMS data),^40^ reducing NRMSE by factors of 5‐6. Navigator‐based and navigator‐free phase corrections achieve comparable reconstruction fidelity, with slightly higher error in the navigator‐free reconstruction (see inset values in Figure 3). The strong artifacts in the navigator‐free SMS‐DAGER reconstruction are mostly located in the background where phase estimates are purely noise. The quality of navigator‐free reconstruction within the brain suggests the initial SENSE reconstruction provides a good estimation of the phase errors (for brain region).

### 4.3 In vivo in‐plane acceleration

Supporting Information Figure S4 shows in‐plane acceleration results from subject 1. Conventional SENSE at R = 6 suffers from noise amplification and residual aliasing even with 32 coil elements. DAGER reconstruction is considerably improved. As in simulation, phase‐error correction with and without an explicitly acquired phase navigator demonstrate very similar performance. Therefore, the navigator‐free method was adopted for the rest of this work to reduce specific absorption rate. However, for very high undersampling factors, navigator‐based approach and advanced reconstruction methods might be needed for phase‐error correction (Supporting Information Discussion S2).

One indirect way to characterize dMRI data quality is through the estimation of secondary fiber populations within a voxel, where automatic relevance determination is used for deciding if a secondary fiber is supported by the data.^35^ Detection of multiple fibers requires both high contrast‐to‐noise ratio and high angular resolution.

**Figure 4:**
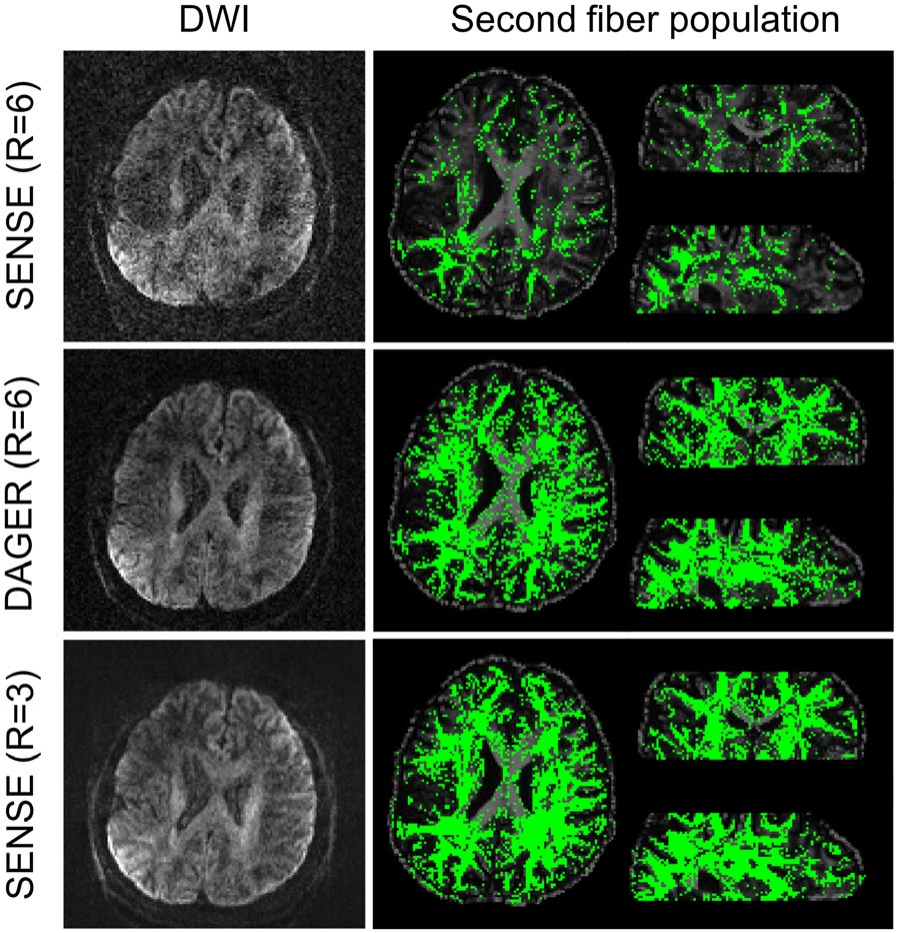
Multiple fiber populations within a single voxel are estimated from R = 6 data reconstructed with SENSE (top) and DAGER (middle), and R = 3 data reconstructed with SENSE (bottom). The R = 3 data are used as a reference here. The second fiber populations for R = 3 data, DAGER (R = 6) data, and SENSE (R = 6) data are 89355, 78867, and 23645, respectively. The same threshold (0.05) is used for all data sets

As shown in Figure 4, SENSE reconstruction at R = 6 fails to capture many secondary fiber populations, likely due to the low contrast‐to‐noise ratio . In this same data, secondary fibers are recovered throughout the brain in the DAGER reconstruction. This indicates DAGER reconstruction has high contrast‐to‐noise ratio and no excessive angular blurring.^49^, ^50^ Receiver operating characteristic (ROC) analysis also demonstrates a greater capacity in capturing second fiber populations using DAGER compared with SENSE (Supporting Information Figure S5). The reference SENSE reconstruction of R = 3 data provides a good CNR with negligible residual artifacts.

### 4.4 In vivo SMS with in‐plane acceleration

**Figure 5:**
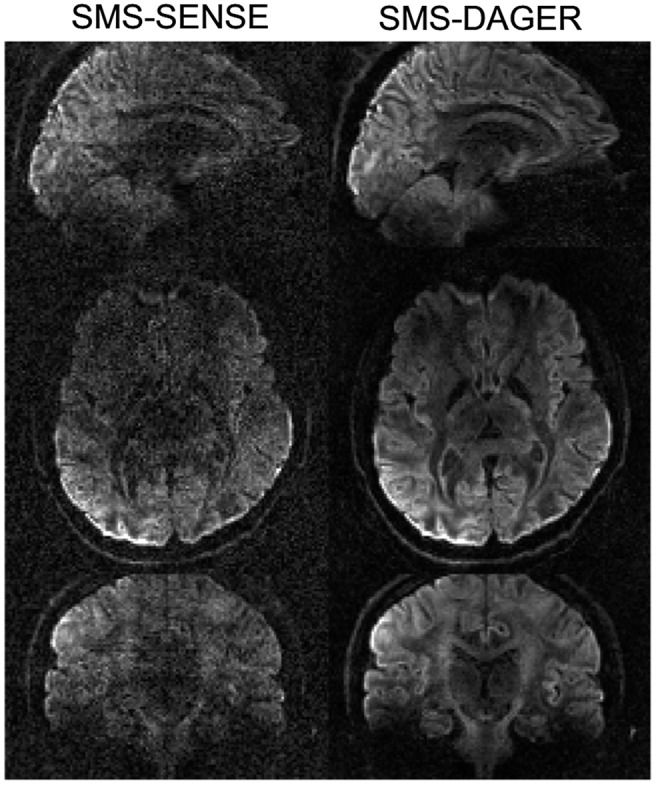
Reconstruction of the same in vivo SMS data using SMS‐DAGER and SMS‐SENSE, with total acceleration factor 12 (MB = 4 and R = 3)

As shown in Figure 5, conventional SENSE reconstruction suffers from amplified noise due to the high acceleration applied in both through‐plane and in‐plane. From the same data, DAGER reconstructs high‐quality images with suppressed noise and aliasing artifacts. As shown in Supporting Information Figure S6, the SMS‐DAGER reconstruction demonstrate consistent contrast with the SB reference (SB‐3ave), which is 3 repetitions averaged. In addition, the noise level of the SMS image is similar to the SB reference despite a 3‐fold scan time reduction, suggesting the GP prior can effectively reduce noise by exploiting the common features between diffusion volumes.

**Figure 6:**
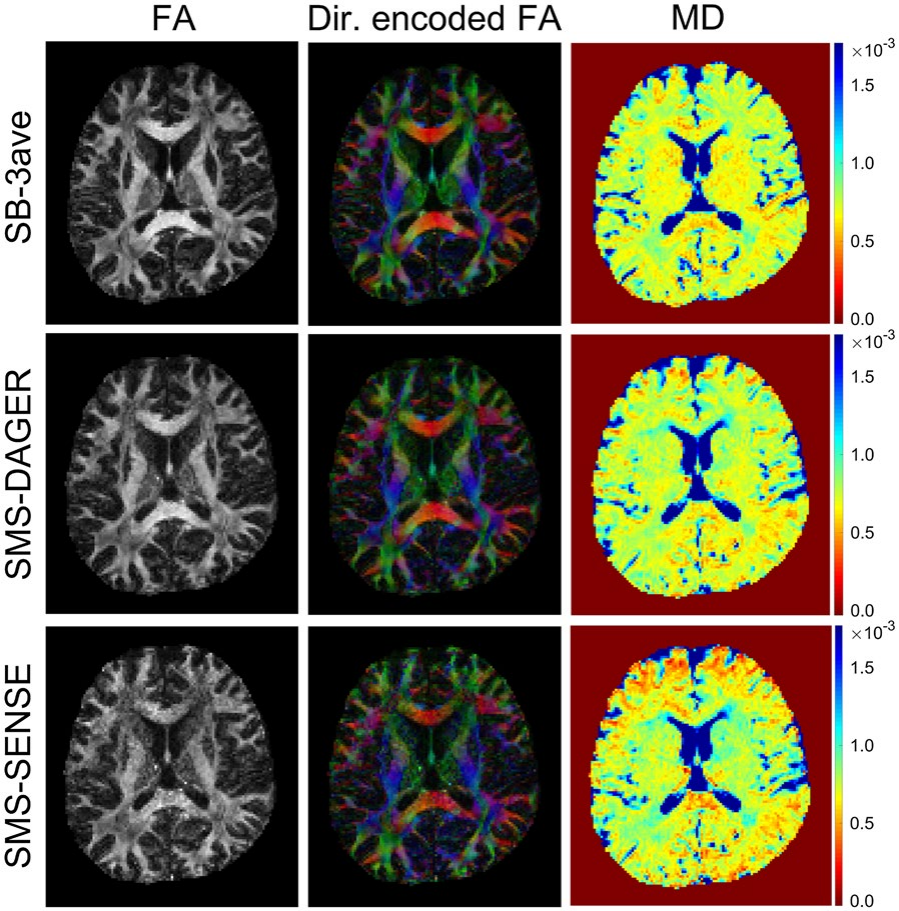
Diffusion tensor analysis of subject 4 data. Comparison of SMS data (MB = 4 and R = 3) reconstructed using both SENSE and DAGER, and a high‐SNR SB reference (SB‐3ave). Three parametric maps are shown here: fractional anisotropy (FA), direction‐encoded colormap (red: right‐left; green: anterior‐posterior; blue: superior‐inferior) and mean diffusivity (MD)

Diffusion tensor fits to SMS data in subject 4 are shown in Figure 6. Tensor metrics derived from SMS‐DAGER reconstruction are consistent with the high‐SNR SB‐3ave reference, while the conventional SENSE reconstruction of the same data gives a noisy estimation of fractional anisotropy and lower mean diffusivity values, likely due to the low image SNR and residual aliasing artifacts.

### 4.5 Evaluation of angular resolution

**Figure 7:**
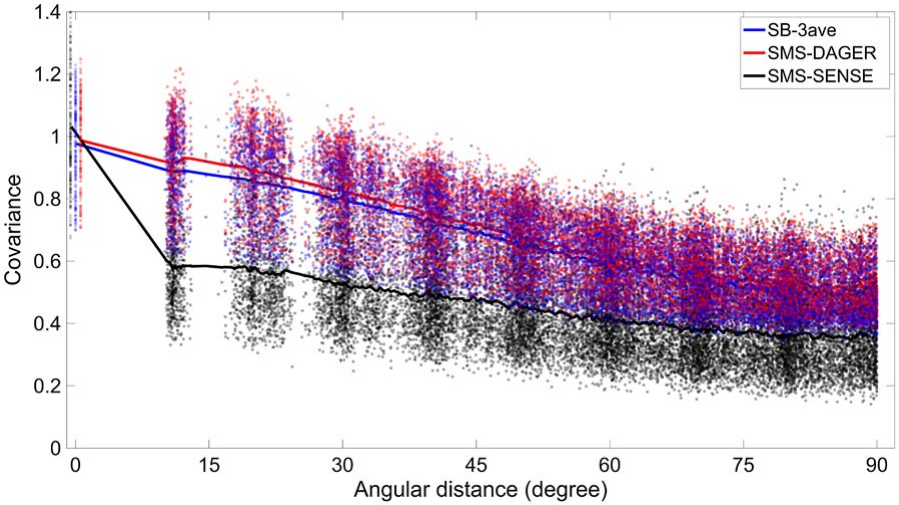
Signal covariance of dMRI images for high‐SNR SB reference (SB‐3ave) and SMS data (MB = 4, R = 3) reconstructed using SMS‐DAGER and SMS‐SENSE. Each point represents 1 diffusion direction. The averaged covariances are plotted as solid lines. For each data set, the covariance is normalized by the median of signal variances (angular distance = 0°). Note that in the plot we deliberately shift signal‐variance points by a small amount along the angular‐distance axis for better visualization of the differences

In Figure 7, the covariance of the dMRI signal between 2 images is plotted as a function of the angle between them in q‐space (points with 0° angular distance are the signal variance of a single volume). Angular smoothing would lead to increased covariance at large angular difference. Compared with the high‐SNR SB‐3ave reference, SMS‐DAGER images have only slightly higher covariance, indicating preservation of angular resolution in the DAGER reconstruction. SMS‐SENSE reconstruction of the same data is corrupted by noise, giving low covariance between diffusion directions.

**Figure 8:**
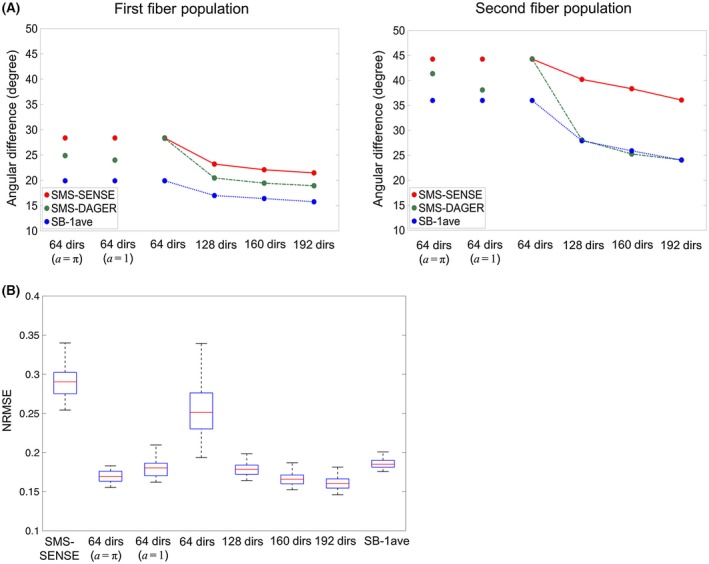
A, Evaluation of fiber orientations in subject 4, using 2 averages from the SB data (SB‐2ave) as a reference. The angular difference to SB‐2ave is estimated for SB‐1ave (the third SB data set in this subject), SMS‐DAGER and SMS‐SENSE. Angular estimates are separately considered for the first (left) and second (right) fiber population. Four diffusion direction sets are evaluated, which contain 64, 128, 160, and 192 directions, respectively. For the 64‐direction data set, 2 additional DAGER reconstructions with enforced smoothness (*a* = 1 and *a* = π) are also applied (The three 64‐direction estimates are identical for SMS‐SENSE, and for SB1‐ave). For clear visualization of the differences between methods, error bars are not included here. B, Box‐Whisker plot of NRMSE for different reconstructions. The red line on each box indicates the median NRMSE for all diffusion directions, and the bottom/upper edges indicates the 25^th^/75^th^ percentiles

Comparison of fiber orientations, i.e., the deviation from that estimated from the SB‐2ave data, is shown in Figure 8. For a large number of diffusion directions (128+), the angular differences derived from the SMS‐DAGER images are comparable to those derived from the SB‐1ave images, with slightly worse performance for the first fiber and equivalent performance for the second fiber (*P* > 0.01, see Supporting Information Table S1 for details). This suggests preservation of SNR and angular resolution in DAGER reconstruction. The fiber orientations derived from the SMS‐SENSE data deviate significantly from the reference, even with many diffusion directions, which is likely due to the noise amplification and residual artifacts in the images.

The number of diffusion directions is a key determinant of the quality of fiber orientation estimation. It limits the number of diffusion volumes that are available to our local GP estimation and constrains the angular resolution that can be resolved using dMRI. With only 64 directions, SMS‐DAGER performs similarly as SMS‐SENSE, presumably reflecting that q‐space samples are too far apart to provide useful information for local GP estimation. We can explore the effect of the smoothness hyper‐parameter *a* for this 64‐direction data set. If we fix *a* to an inflated value, the images have higher SNR, suggesting improved reconstruction conditioning (see Supporting Information Figure S7). However, this enforced‐smoothness constraint may introduce angular blurring beyond the intrinsic signal smoothness. As shown in Figure 8A, with a slight smoothness constraint (*a* *= *1), the estimation of angular resolution is improved. However, when a strong smoothness constraint (*a = *π) is applied, the angular difference inflates, suggesting a loss of angular resolution in the reconstruction. Indeed, it is noticeable that the image contrast for a single image with *a = *π (indicative of shared information over the entire q sphere) is very similar to a mean diffusivity weighting (Supporting Information Figure S7).

NRMSE of DAGER reconstruction increases as the number of directions decreases (Figure 8B), which is consistent with the fiber orientation evaluation. Enforced smoothness (fixed *a*) improves SNR and reduces NRMSE, but it compromises angular resolution (Figure 8A). The NRMSE of SB‐1ave data provides a useful reference point for this kind of comparison against a reference data set with noise and potential misregistration. SMS‐SENSE reconstruction gives the worst result with highest NRMSE.

### 4.6 Diffusion tractography

**Figure 9:**
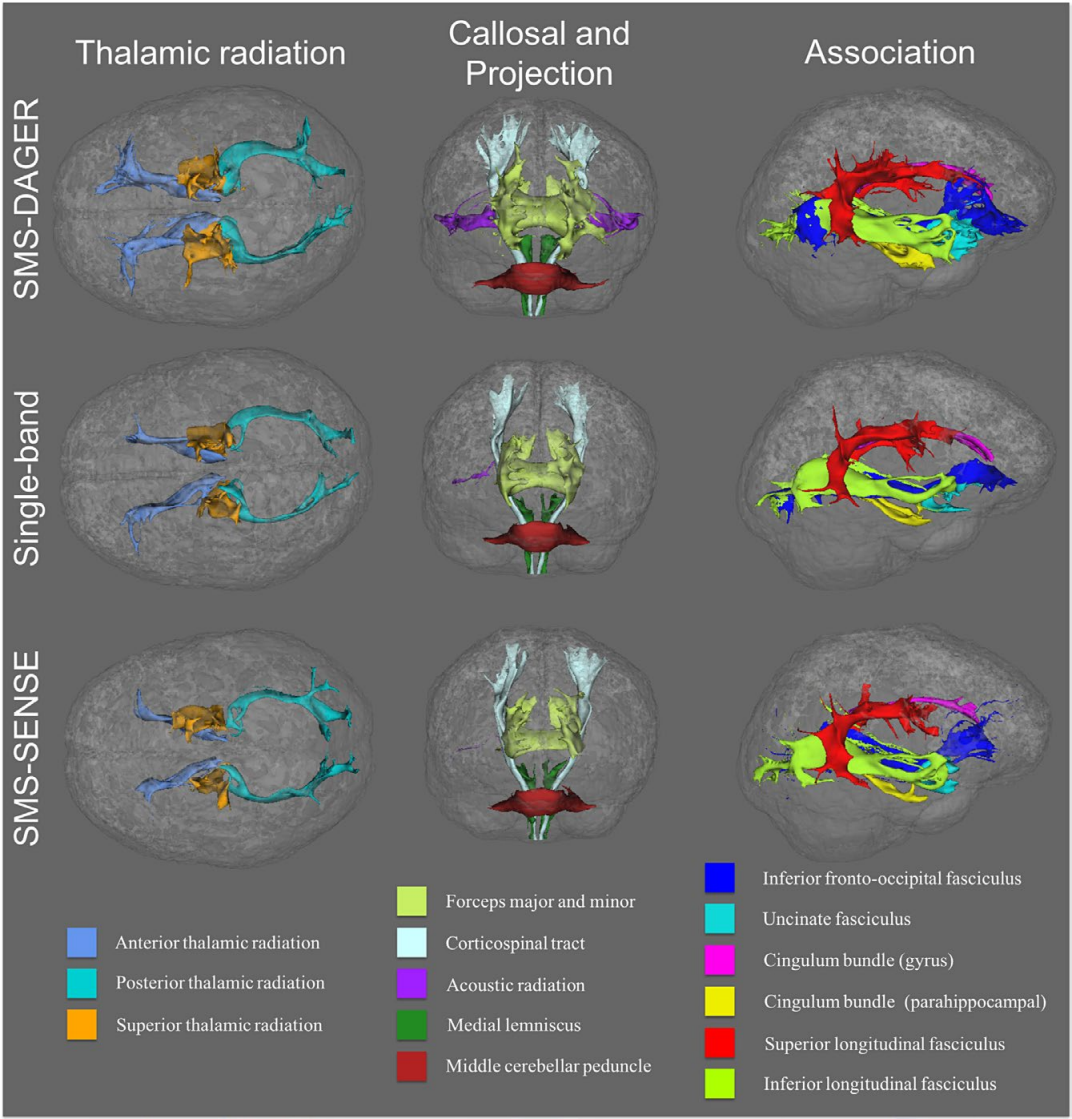
White matter fiber tracts generated using AutoPtx. 14 major white matter pathways (constituting 27 separate tracts in right and left hemispheres) are rendered in superior (left, thalamic radiation), anterior (middle, projection and callosal fibers) and lateral (right, association fibers) views. To match the scan time, SMS data and SB data contain 192 and 50 directions, respectively. SMS data reconstructed with DAGER support the delineation of all tracts, whereas the SB data and the SMS data reconstructed with SENSE fail to capture some tracts. Most notable are the acoustic radiations (missing from both SB and SMS‐SENSE reconstructions)

We conducted automatic mapping of 14 major white matter tracts using AutoPtx^47^ on the full‐FOV data sets in subjects 1‐3. To match the scan time (~11 min), SMS‐ and SB‐data were acquired with 192 and 50 directions, respectively. As shown in Figure 9, all the fiber bundles are robustly identified from the SMS‐DAGER data. The SB results capture many fiber bundles, but its performance is compromised by the limited number of diffusion directions achievable in this scan time. Tracts are generally less abundant in the SB and SMS‐SENSE data than the SMS‐DAGER data. In some cases, tracts are totally missing at the rendering threshold. For example, the acoustic radiation tracts (Figure 9, middle column, in purple) are clearly captured by the SMS‐DAGER data, but essentially absent in both the SB and the SMS‐SENSE results. Note these renderings used the same threshold across the different data sets but required these to be determined manually. However, where tracts are largely absent, no threshold would produce decent tract reconstructions, and the overall conclusions about the relative performance of the data sets does not change with threshold level. These results demonstrated improvement of DAGER in a domain that is important in practice (beyond producing high‐quality image).

## 5 DISCUSSION

This work aims to address one of the major limitations for SMS acceleration: the ill‐conditioned reconstruction at high acceleration factor, which usually results in a strong noise penalty. Improved sampling schemes such as CAIPIRINHA^51^ can mitigate this issue, but it remains challenging for dMRI due to its intrinsically low SNR, particularly in combination with in‐plane acceleration to reduce distortion and blurring. This limitation was specifically noted in the design of the HCP acquisition at 3T, where in‐plane acceleration was abandoned due to significant SMS image degradation.^33^ DAGER is a new approach for improving SMS through a k‐q reconstruction that leverages the redundancy of spatial information in q‐space.

In DAGER, we extend conventional undersampling into k‐q space by using different sampling patterns within a local q‐space neighborhood. These sampling patterns are produced by shifting a 3D‐EPI trajectory along both k_y_ and k_z_ directions (Figure 1B). This benefits SMS by providing complementary k‐space information along both k_y_ and k_z_ directions. With a maximum scan time, DAGER will allow one to achieve higher acceleration (including higher SMS factor) and thus get more q‐space samples (which in turn increase the information available to GP). In comparison to previous work that accelerates purely in q‐space (e.g., compressed sensing), k‐q acceleration can reduce distortion/blurring with in‐plane acceleration and increase SNR‐efficiency (where optimal TR = 1‐3 s, depending on field strength).^52^ Using the DAGER approach, we have demonstrated highly accelerated imaging with superior performance compared with conventional parallel imaging, achieving high SMS acceleration (MB = 4) with in‐plane undersampling (R = 3). Alternatively, DAGER can be used to increase acceleration in 1 dimension: this was demonstrated here with high in‐plane accelerations, but may also enable higher MB factors.

Diffusion in biological tissue ranges from free to restricted diffusion, causing the observed diffusion signal in q‐space to be relatively smooth. This property has been exploited in GPs for data analysis.^23^, ^25^ Unlike methods used in other k‐q reconstructions^19^, ^21^ that aim to find a parametric model for the diffusion function, the GP method aims to find a parametric model for the covariance function, with the hyper‐parameters related to the signal smoothness in q‐space learned from the data itself.^22^, ^24^ DAGER uses an iterative updating scheme to calculate hyperparameter estimates and image reconstruction. This differs from classical model fitting where the model is identical in each iteration. Nevertheless, we find the reconstruction typically converges after 10‐15 iterations (Supporting Information Discussion S3).

The optimal hyper‐parameters could depend on tissue type, suggesting it might be preferable to estimate them separately for, e.g., gray and white matter. Such a situation would add practical complication to DAGER due to the need for tissue‐type segmentation. Fortunately, previous investigations of this found no evidence for overfitting of this kind.^24^ In addition, the GP approach provides an elegant way to model multishell dMRI data, allowing the estimation of data across shells and directions.^24^ Future work will expand DAGER to multiple q‐space shells, enabling more sophisticated analyses for microstructural information, such as NODDI.^53^ Finally, the consistent use of GPs in both data reconstruction and preprocessing raises the possibility of integrating the 2 procedures, a synergy which could further improve dMRI results.

As DAGER pulls information from adjacent q‐space locations, it could introduce angular smoothing if the hyper‐parameters overestimate the actual signal smoothness. Figure 8A shows a case where a strong, fixed smoothness constraint led to the loss of angular resolution. We investigated the effects of DAGER reconstruction on angular resolution. First, we compared the covariance of reconstructed images with respect to q‐space angular distance, finding that the signal covariance derived from the DAGER result is consistent with that from high‐SNR reference data (Figure 7), suggesting the estimated hyper‐parameters are able to capture the underlying covariance of in vivo dMRI signal.

Second, we investigated the effects of DAGER reconstruction on voxel‐wise fiber orientations compared with a reference. For more than 128 directions, angular differences between DAGER and the high‐SNR reference are not considerably larger than those derived from the conventional SB data (Figure 8A); with a small number of directions (e.g., 64), DAGER reconstruction is very close to SENSE (Supporting Information Discussion S4). NRMSE evaluation of DAGER reconstruction (Figure 8B) also reveals that the reconstruction performance depends on the number of directions, with reduced benefit of DAGER when few directions are used. These evaluations and investigations suggest DAGER reconstruction can provide a good preservation of angular resolution.

The calibration of noise variance can affect the reconstruction performance, especially for low SNR data (see Supporting Information Figure S3), which are common in dMRI. In simulation, DAGER’s noise variance estimates were demonstrated to be close to the ground truth, but more importantly the image errors (NRMSE) using the estimated noise variance were very similar to that of a reconstruction with the correct noise variance. In addition, the automatic determination of reconstruction parameters is pragmatic, as no manual adjustment is needed, unlike many previous k‐q methods. Noise in magnitude images follows a Rician distribution, which can be approximated by a Gaussian distribution if the data have sufficient SNR. For low‐SNR dMRI data (e.g., high resolution, high b values), this approximation will be inaccurate and can bias estimates.^54^ This problem could be addressed by estimating i.i.d. Gaussian noise in k space data and using a non‐Gaussian likelihood for the image‐space hyper‐parameters.^22^

For a fixed number of diffusion directions, we expect a limitation on the acceleration factor that can be achieved. However, the achievable acceleration factor will depend on many additional factors, including receive coil, field strength, b value, radiofrequency pulses, k‐q sampling, and spatial resolution. It is also expected that different analysis methods and models will vary in their sensitivity to noise amplification and angular smoothness. As such, it is not straightforward to determine an appropriate acceleration factor without resorting to some empirically determined heuristics that likely need to be based on the final outputs of the target data analysis.

Although the current work only demonstrates accelerated 2D and 2D‐SMS dMRI, other acquisition methods like 3D multislab should also be compatible with DAGER, provided a variable k‐q sampling scheme is incorporated. Recently, several studies have demonstrated simultaneous multislab acquisition for dMRI, enabling optimal SNR efficiency with shorter scan time.^55^, ^56^ It is expected the combination of DAGER and simultaneous multislab acquisition can achieve even higher scan efficiency with improved image quality.

Finally, parallel imaging reconstruction is a mature field, with a broad range of techniques from SENSE to generalized auto‐calibrating partially parallel acquisition (GRAPPA)^4^, ^5^, ^40^, ^41^, ^57^ and many regularization approaches,^6^, ^7^, ^8^, ^9^, ^10^ which can potentially be embedded within the DAGER framework for further improvement, particularly at the limits of the current DAGER implementation (e.g., at high acceleration or with a reduced number of diffusion directions). However, the primary goal of this study is to demonstrate that sharable information in q‐space can be leveraged to improve upon existing parallel imaging reconstructions. Exploration of how well different regularization techniques can take advantage of this shared information will be the topic of future work.

## 6 CONCLUSIONS

In this work, we developed a method to accelerate dMRI acquisition, which incorporates sharable information between q‐space samples to improve the reconstruction of k‐space data. This k‐q reconstruction approach uses GPs to estimate and exploit local smoothness without introducing undesirable angular blurring. Results from simulation and in vivo data demonstrate the efficacy of the proposed method, particularly for high SMS acceleration with in‐plane undersampling.

## Supporting information


**FIGURE S1** Left: An L‐curve shows the data fidelity term (${\left\|\mathrm{Au}-d\right\|}_{2}^{2}$) and the regularization term (${\left\|u\right\|}_{2}^{2}$) for different regularization parameters used in the reconstruction of in vivo dMRI data. From left to right, the images correspond to regularization parameters that are too small (noisy images), L‐curve optimal and too large (aliased images). The regularization term at the “corner” ($\lambda_{3}$) provides an acceptable compromise between the two error metrics, which is used for SENSE and SMS‐SENSE reconstruction. To achieve minimal aliasing artifacts, a lower regularization parameter ($\lambda_{2}$) is used for phase error estimation and DAGER initialization. Right: SMS‐SENSE reconstructed images with different regularization parameters as shown in the L‐curve
**FIGURE S2** Effects of k‐q sampling and phase error correction on DAGER reconstruction of simulated data. Two data sets are simulated with R = 6: one containing no phase errors and acquired using a fixed k‐q sampling (k‐space sampling pattern is identical for all q‐space points); the other containing phase errors and acquired using the variable k‐q sampling (different k‐space sampling patterns are used within local neighborhood in q‐space). Top row: the data set with fixed k‐q sampling but without phase errors is reconstructed using SENSE and DAGER. Bottom row: the data set with variable k‐q sampling and simulated phase errors is reconstructed using DAGER with and without phase error corrections. The ground truth image is shown on the left. The difference images are shown next to the reconstructed images, which are scaled by a factor of 3, except for the bottom right, which is scaled by a factor of 10. If a fixed k‐q sampling is used, DAGER cannot correctly recover the image even if it contains no phase errors (top‐right). For data with phase errors, DAGER without phase error correction contains significant image artifacts (bottom left). With the variable k‐q sampling and phase error correction, DAGER provides a good reconstruction with minor residual artifacts (bottom right)
**FIGURE S3** Simulations were performed at different levels of added noise (SNR = 6‐60) with undersampling factor R = 6 and 128 directions. The noise variance estimated by DAGER are indicated by the solid circles. The lines indicate reconstruction NRMSE when k‐space noise variance levels are fixed in the range $\sigma_{\text{test}}^{2}=$(2^‐10^ ‐ 2^10^)×$\sigma_{\text{true}}^{2}$. The NRMSE values are averaged over all diffusion directions and the binary logarithm is shown here for ease of visualization. As shown in the figure, all plots have an area of low NRMSE for test noise variances close to the ground‐truth variance $\sigma_{\text{true}}^{2}$ (log_2_ ($\sigma_{\text{test}}^{2}/\sigma_{\text{true}}^{2}$) = 0), suggesting that the reconstruction fidelity is not overly sensitive to the estimate of this hyper‐parameter, particularly if the true noise variance is low (i.e., SNR = 30 and 60). For low SNR data, deviation from the true variance by more than a factor of ~3 can lead to a sharp increase of reconstruction errors (i.e., SNR = 6 and 10). Most importantly, the DAGER enables noise variance estimates (solid circles) that provide similar or better NRMSE as compared with the true noise variance, enabling a robust reconstruction
**FIGURE S4** Reconstruction of in‐plane undersampled data acquired from subject 1. The R = 6 data is reconstructed with SENSE and DAGER (with and without navigator acquisition)
**FIGURE S5** Comparison of ROC curves for second fiber identification using DAGER(R = 6) data and SENSE(R = 6) data. SENSE (R = 3) result is used as a reference. Threshold values between 0 and 0.5 (0.01 step size) are tested. The ROC curve of DAGER is closer to the upper left corner compared with the ROC curve of SENSE, demonstrating a greater capacity in capturing second fiber population using DAGER
**FIGURE S6** Comparison of SMS‐DAGER reconstruction (top) and high‐SNR single‐band reference (bottom), generated by averaging three single‐band data sets (SB‐3ave), in subject 4. Four diffusion directions of a transverse slice are shown here. The single‐band protocol acquired 21 slices in ∼33min, whereas the SMS‐DAGER images acquired 84 slices (whole brain) in ∼11min
**FIGURE S7** Reconstruction with 64 diffusion directions using SMS‐SENSE and three SMS‐DAGER configurations, where smoothness hyper‐parameter *a* is estimated from the data (0.7) and manually set to 1 and π, respectively
**TABLE S1** Analysis of fiber orientation estimations based on the SB‐1ave data and the SMS‐DAGER data. The SB‐2ave data is used as a reference here. The absolute angular difference tells how much SB‐1ave or SMS‐DAGER data deviate from the reference in the estimation of fiber orientations. The deviations for these two data sets are compared and tested
