# Supplementary material for: Diffusion Acceleration with Gaussian process Estimated Reconstruction (DAGER)
Source: Magn Reson Med. 2019 Mar 1;82(1):107–25. doi: 10.1002/mrm.27699 (PMC6492188; doi:10.1002/mrm.27699)
Supplement: Supplementary file 1 — FIGURE S1 Left: An L‐curve shows the data fidelity term (‖Au-d‖22) and the regularization term (‖u‖22) for different regularization parameters used in the reconstruction of in vivo dMRI data. From left to right, the images correspond to regularization parameters that are too small (noisy images), L‐curve optimal and too large (aliased images). The regularization term at the “corner” (λ3) provides an acceptable compromise between the two error metrics, which is used for SENSE and SMS‐SENSE reconstruction. To achieve minimal aliasing artifacts, a lower regularization parameter (λ2) is used for phase error estimation and DAGER initialization. Right: SMS‐SENSE reconstructed images with different regularization parameters as shown in the L‐curve FIGURE S2 Effects of k‐q sampling and phase error correction on DAGER reconstruction of simulated data. Two data sets are simulated with R = 6: one containing no phase errors and acquired using a fixed k‐q sampling (k‐space sampling pattern is identical for all q‐space points); the other containing phase errors and acquired using the variable k‐q sampling (different k‐space sampling patterns are used within local neighborhood in q‐space). Top row: the data set with fixed k‐q sampling but without phase errors is reconstructed using SENSE and DAGER. Bottom row: the data set with variable k‐q sampling and simulated phase errors is reconstructed using DAGER with and without phase error corrections. The ground truth image is shown on the left. The difference images are shown next to the reconstructed images, which are scaled by a factor of 3, except for the bottom right, which is scaled by a factor of 10. If a fixed k‐q sampling is used, DAGER cannot correctly recover the image even if it contains no phase errors (top‐right). For data with phase errors, DAGER without phase error correction contains significant image artifacts (bottom left). With the variable k‐q sampling and phase error correction, DAGER provides a good reconstruction [file MRM-82-107-s001.pdf]

Supporting Information Methods

Supporting Information Methods S1: Sequence optimization to reduce SAR for SMS-dMRI at 7T

First, RF pulses were designed to be low SAR. Excitation used a conventional MB pulse and refocusing used a MultiPINS pulse (Eichner et al., MRM 2014), both with TBWP = 2.52 and duration = 10.9ms. Second, the diffusion module used a modified monopolar preparation (single 180° pulse), in which part of the second diffusion gradient is negated and placed before the 180° pulse to reduce eddy currents. Finally, it was found that the initial SENSE reconstruction was able to provide a good estimation of the phase errors, enabling us to eliminate the navigator for SMS acquisitions, thereby removing one RF refocusing pulse. The final sequence for the MB=4 acquisition had low SAR level (<50% of scanner limits).

Supporting Information Discussion

Supporting Information Discussion S1: Multi-shot EPI acquisition for b=0 data

For this proof-of-principle study, we focus on the reconstruction of dMRI data and acquire b=0 data using a multi-shot EPI protocol that samples full k-space to avoid strong reconstruction artifacts at high acceleration. However, the multi-shot sequence will increase the sensitivity to subject motion if the acquisition time is too long, even for b=0 data without significant shot-to-shot phase errors. This issue could be mitigated by sampling partial k space (reducing the number of segments), using lower slice acceleration (e.g. MB=2) and/or acquiring single-band b=0 data.

Supporting Information Discussion S2: Navigator-free approach for phase error correction

Another limit to acceleration with DAGER relates to the need to estimate and account for motion-induced phase errors. Our navigator-free approach uses conventional parallel imaging methods to estimate phase errors (Chen et al., NeuroImage 2013). This approach would fail at acceleration levels where parallel imaging is not able to provide an accurate image reconstruction. Advanced reconstruction methods, such as low-rank modelling and compressed sensing, may be able to support higher acceleration factors. Alternatively, DAGER can use explicitly-acquired navigators. At ultra-high field, where SMS acceleration is generally limited by SAR, the need for an additional refocusing pulse for a navigator is

problematic. At lower field, acquisition of navigators is more feasible, incurring a slight loss of efficiency due to longer TR.

Supporting Information Discussion S3: GP hyper-parameter estimation

The hyper-parameters in the GP model are estimated from the data based on a Bayesian formulation. One subtle but important detail is that image aliasing, which the reconstruction aims to remove based on smoothness, has a detrimental effect on hyper-parameter estimation. An iterative updating scheme is adopted in DAGER, where the hyper-parameter estimates and the image reconstruction are consecutively updated such that errors in both procedures are gradually suppressed over multiple iterations.

It is worth noting that this reconstruction scheme differs from classical model fitting procedure where the model is identical in each iteration. Instead, in DAGER reconstruction the model is refined in each iteration, which might lead to a non-monotonically decreasing cost-function. In our experiments, we find the reconstruction typically converges after 10-15 iterations and the model becomes relatively stable afterwards. This is demonstrated in simulation (Fig. 2), where the iteration converges after 13 iterations. However, under certain circumstances, the proposed algorithm might fail to converge, such as strong subject motion, severe eddy current distortion and a low number of diffusion directions. In future work, we aim to integrate the motion and distortion correction, which could aid in convergence under these conditions.

Supporting Information Discussion S4: Effects of the number of diffusion directions on DAGER reconstruction

As DAGER relies on the joint information between q-space neighbours, the number of diffusion directions is expected to affect the reconstruction performance. In this work, we found that DAGER reconstruction with 64 diffusion directions is very close to SENSE reconstruction, indicating low covariance between the available q-space samples (the average angular difference between each point and its nearest neighbour is about 20 degree). This result suggests that a relatively large number of directions are needed for DAGER reconstruction. However, given that DAGER is intended to support higher SMS acceleration factors, the burden of time required to reach a target number of directions goes down, leading

to a synergy between the needs of the GP and the benefits it delivers. Here, we were able to achieve high quality reconstructions by acquiring 128 directions in ~7.5min. Additionally, once distortion correction is incorporated in DAGER, we may be able to exploit q-space symmetry (the equivalence of q-space samples on opposite sides of the sphere) to increase the number of 'effective' q-space neighbours. This will be investigated in future work.

References

Eichner, C., Wald, L.L. and Setsompop, K., 2014. A low power radiofrequency pulse for simultaneous multislice excitation and refocusing. Magnetic resonance in medicine, 72(4), pp.949-958.

Chen Nk, Guidon A, Chang HC, Song AW. A robust multi-shot scan strategy for high-resolution diffusion weighted MRI enabled by multiplexed sensitivity-encoding (MUSE). NeuroImage 2013;72:41-47.

Supporting Information Figures

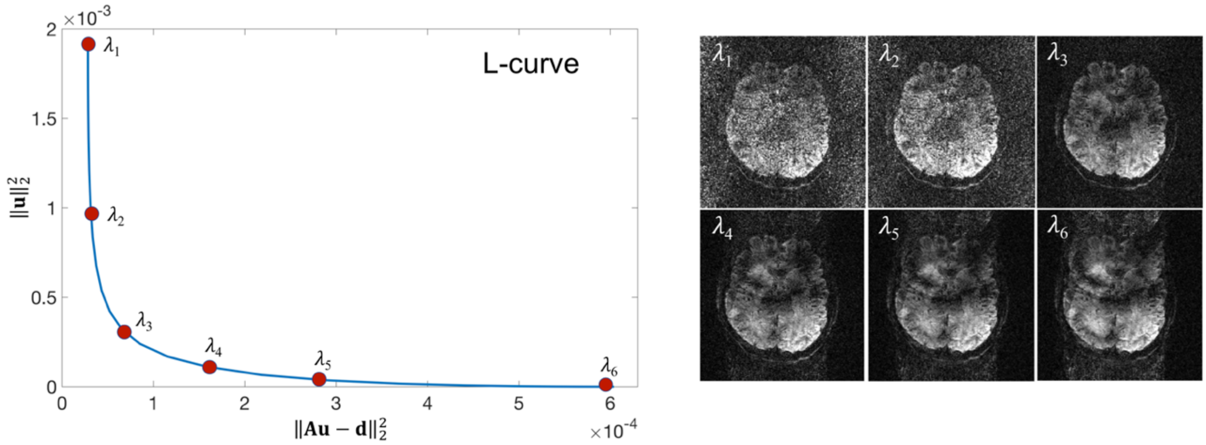

Supporting Information Figure S1. Left: An L-curve shows the data fidelity term ($||\mathbf{Au}-\mathbf{d}||_2^2$) and the regularization term ($||\mathbf{u}||_2^2$) for different regularization parameters used in the reconstruction of in vivo dMRI data. From left to right, the images correspond to regularization parameters (λ) that are too small (noisy images), L-curve optimal and too large (aliased images). The regularization term at the "corner" (λ_3) provides an acceptable compromise between these two error metrics, which is used for SENSE and SMS-SENSE reconstruction. To achieve minimal aliasing artefacts, a lower regularization parameter (λ_2) is

used for phase error estimation and DAGER initialization. Right: SMS-SENSE reconstruction with different regularization parameters as shown in the L-curve.

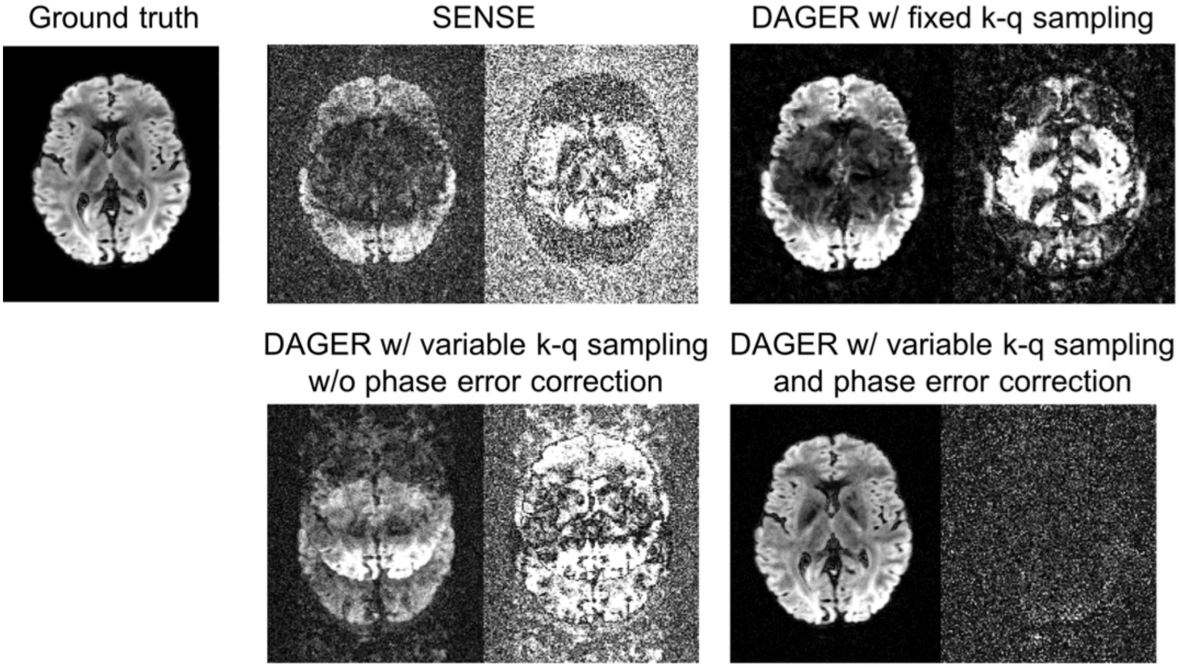

Supporting Information Figure S2. Effects of k-q sampling and phase error correction on DAGER reconstruction of simulated data. Two data sets are simulated with R=6: one containing no phase errors and acquired using a fixed k-q sampling (k-space sampling pattern is identical for all q-space points); the other containing phase errors and acquired using the variable k-q sampling (different k-space sampling patterns are used within local neighborhood in q-space). Top row: the data set with fixed k-q sampling but without phase errors is reconstructed using SENSE and DAGER. Bottom row: the data set with k-q sampling and simulated phase errors is reconstructed using DAGER with and without phase error corrections. The ground truth image is shown on the left. The difference images are shown next to the reconstructed images, which are scaled by a factor of 3, except for the bottom right, which is scaled by a factor of 10. If a fixed k-q sampling is used, DAGER cannot correctly recover the image even if it contains no phase errors (top-right). For data with phase errors, DAGER without phase error correction contains significant image artifcats (bottom left). With the variable k-q sampling and phase error correction, DAGER provides a good reconstruction with minor residual artifacts (bottom right).

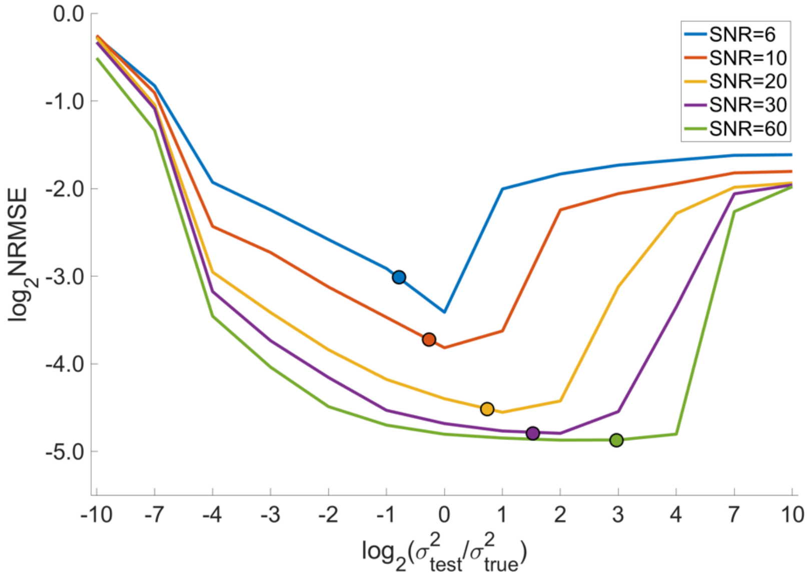

Supporting Information Figure S3. Simulations were performed at different levels of added noise (SNR=6 - 60) with undersampling factor R=6 and 128 directions. The noise variance estimated by DAGER are indicated by the solid circles. The lines indicate reconstruction NRMSE when k-space noise variance levels are fixed in the range σ^2_{test} =(2^{-10} - 2^{10})×σ^2_{true}. The NRMSE values are averaged over all diffusion directions and the binary logarithm is shown here for ease of visualization. As shown in the figure, all plots have an area of low NRMSE for test noise variances close to the ground-truth variance σ^2_{true} ($\log_2(\sigma^2_{\mathrm{test}}/\sigma^2_{\mathrm{true}}) = 0$), suggesting that the reconstruction fidelity is not overly sensitive to the estimate of this hyper-parameter, particularly if the true noise variance is low (i.e. SNR=30 and 60). For low SNR data, deviation from the true variance by more than a factor of ~3 can lead to a sharp increase of reconstruction errors (i.e. SNR=6 and 10). Most importantly, the DAGER enables noise variance estimates (solid circles) that provide similar or better NRMSE as compared to the true noise variance, enabling a robust reconstruction.

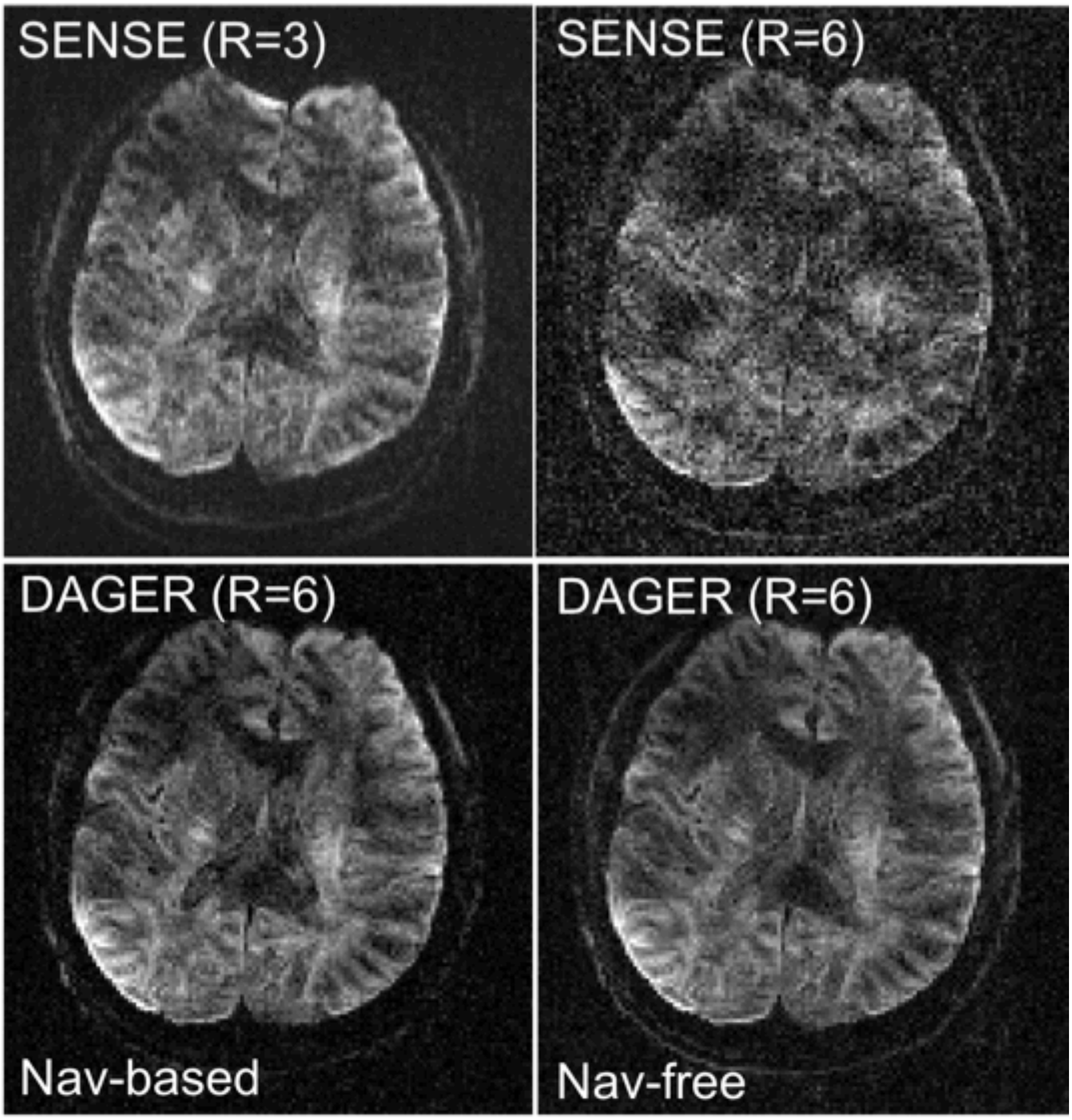

Supporting Information Figure S4. Reconstruction of in-plane under-sampled data acquired from subject 1. The R=6 data is reconstructed with SENSE and DAGER (with and without navigator acquisition).

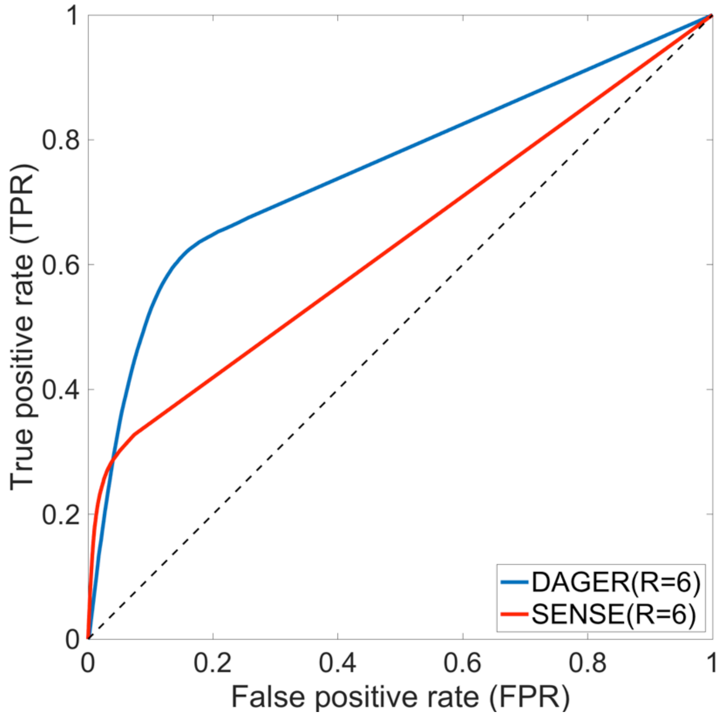

Supporting Information Figure S5. Comparison of Receiver Operating Characteristic (ROC) curves for second fiber identification using DAGER(R=6) data and SENSE(R=6) data. SENSE (R=3) result is used as a reference. Threshold values between 0 and 0.5 (0.01 step size) are tested. The ROC curve of DAGER is closer to the upper left corner compared with the ROC curve of SENSE, demonstrating a greater capacity in capturing second fiber population using DAGER.

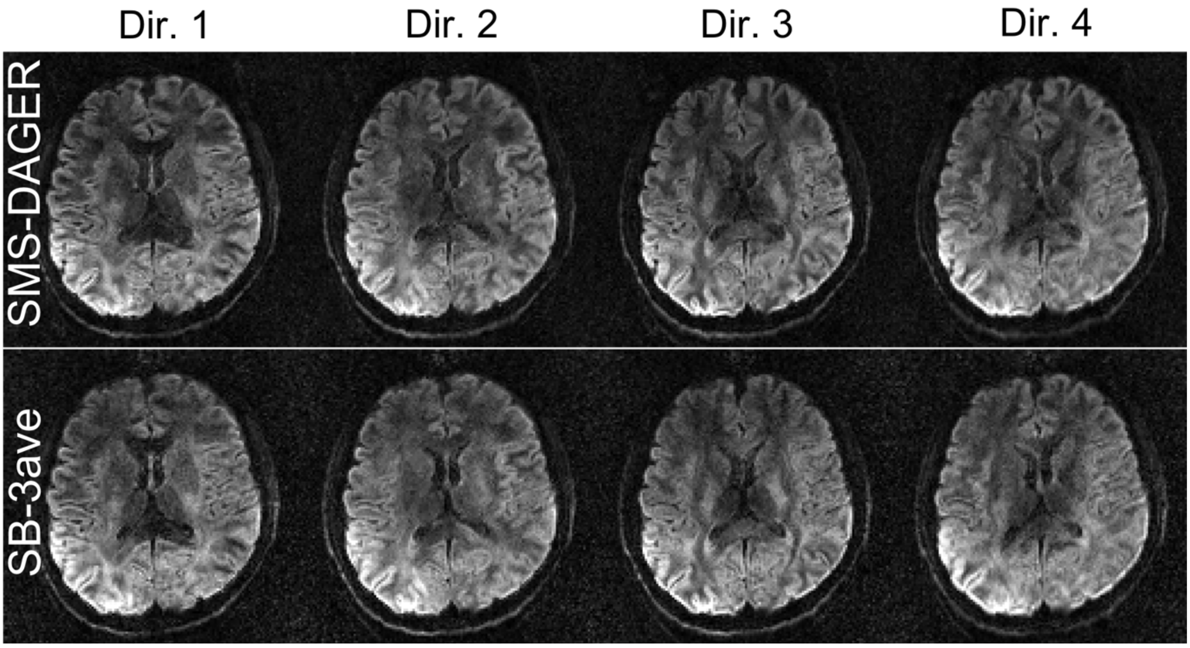

Supporting Information Figure S6. Comparison of SMS-DAGER reconstruction (top) and high-SNR single-band reference (bottom), generated by averaging three single-band data sets (SB-3ave), in subject 4. Four diffusion directions of a transverse slice are shown here. The single-band protocol acquired 21 slices in ~33min, whereas the SMS-DAGER images acquired 84 slices (whole brain) in ~11min.

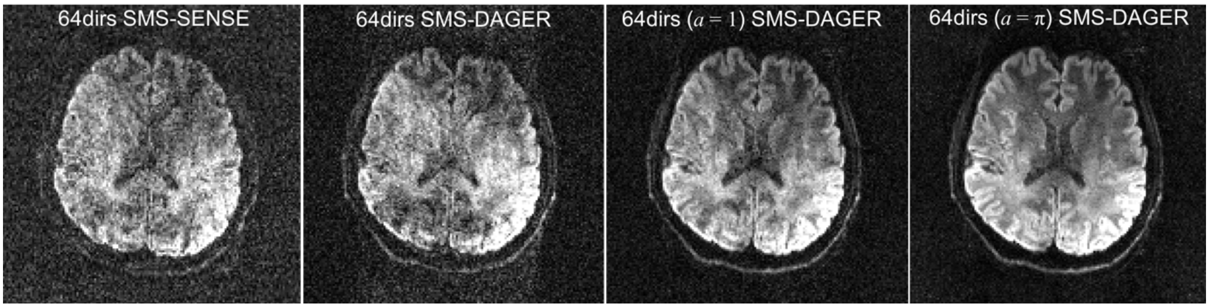

Supporting Information Figure S7. Reconstruction with 64 diffusion directions using SMS-SENSE and three SMS-DAGER configurations, where smoothness hyper-parameter a is estimated from the data (0.7) and manually set to 1 and π, respectively.

	Fiber 1				Fiber 2			
	SB-1ave<br><br>Mean(std)	SMS-DAGER<br><br>Mean(std)	Mean difference<br><br>[95% C.I.]	P value	SB-1ave<br><br>Mean(std)	SMS-DAGER<br><br>Mean(std)	Mean difference<br><br>[95% C.I.]	P value
192 dir.	15.76 (13.53)	18.93 (14.21)	3.17 [2.90,3.43]	<0.001	24.04 (18.05)	24.07 (16.43)	0.03 [-0.45,0.52]	0.890
160 dir.	16.40 (13.9)	19.44 (14.66)	3.04 [2.76,3.31]	<0.001	25.90 (19.02)	25.26 (17.03)	-0.64 [-1.14,-0.12]	0.014
128 dir.	16.99 (14.42)	20.46 (15.74)	3.47 [3.18,3.75]	<0.001	27.91 (19.78)	28.06 (18.29)	0.15 [-0.39,0.68]	0.590
64 dir.	19.91 (16.31)	28.30 (19.51)	8.39 [8.04,8,73]	<0.001	35.98 (21.78)	44.32 (21.59)	8.34 [7.73,8,95]	<0.001
64 dir. (a = 1)	19.91 (16.31)	24.00 (17.73)	4.09 [3.76,4.40]	<0.001	35.98 (21.78)	38.08 (21.41)	2.10 [1.49,2.71]	<0.001
64 dir. ($a = \pi$)	19.91 (16.31)	24.89 (18.49)	4.98 [4.64,5.30]	<0.001	35.98 (21.78)	41.35 (21.82)	5.37 [4.75,5.98]	<0.001

Supporting Information Table S1. Analysis of fiber orientation estimations based on the SB-1ave data and the SMS-DAGER data. The SB-2ave data is used as a reference here. The absolute angular difference tells how much SB-1ave or SMS-DAGER data deviate from the reference in the estimation of fiber orientations. The deviations for these two data sets are compared and tested.
